# Supplementary material for: Impact of the COVID-19 pandemic on medical education: Medical students’ knowledge, attitudes, and practices regarding electronic learning
Source: PLoS One. 2020 Nov 25;15(11):e0242905. doi: 10.1371/journal.pone.0242905 (PMC7688124; doi:10.1371/journal.pone.0242905)
Supplement: S1 Table — (DOCX) [file pone.0242905.s001.docx]

**S1 Table. Distribution of Medical Students according to Medical Schools**

| **Name of the university** | **Frequency** | **Percent** |
| --- | --- | --- |
| University of Tripoli | 1199 | 35.8 |
| Az Zawia University | 497 | 14.8 |
| University of Benghazi | 448 | 13.4 |
| Misrata University | 250 | 7.5 |
| Al-Mergib University | 166 | 5 |
| Sabratha University | 109 | 3.3 |
| Sebha University | 100 | 3 |
| Al-Asmariya University | 91 | 2.7 |
| Omar Al-Mukhtar University | 81 | 2.4 |
| University of Gharyan | 66 | 2 |
| Tobruk University | 50 | 1.5 |
| The Libyan International Medical University | 20 | 0.6 |
| Other | 258 | 7.7 |
