# Supplementary material for: Impact of the COVID-19 pandemic on medical education: Medical students’ knowledge, attitudes, and practices regarding electronic learning
Source: PLoS One. 2020 Nov 25;15(11):e0242905. doi: 10.1371/journal.pone.0242905 (PMC7688124; doi:10.1371/journal.pone.0242905)
Supplement: S1 File — (DOCX) [file pone.0242905.s002.docx]

Impact of COVID-19 on Medical Education in Libya

We welcome your participation in this initiative which aims to assist in the development of medical education in Libya, we are trying to find solutions for difficulties facing medical students in our country at this time.

We require no personal information and all your data shall be encrypted for safekeeping; all we require is your personal opinion which shall be used to find ways to develop our medical education.

Completion of this questionnaire is deemed a consent to participate in this study.

Please sign

- **Gender**: Male Female
- **Age:** __________________
- **Social Status:** Single Married
- **Have you suffered from any financial problems related to the pandemic?**

Yes No

- **Do you currently work in a field unrelated to your medical profession to pay for your learning or training?**
- Yes
- No
- **City where you live**___________________________________________
- **I am a** …………………………. **medical student**
- **Faculty where you study:**
- Tripoli Faculty of Medicine
- Benghazi Faculty of Medicine
- Misurata Faculty of Medicine
- Alzawiyah Faculty of Medicine
- Ghiryan Faculty of Medicine
- Subrata Faculty of Medicine
- Tubruk Faculty of Medicine
- The Libyan International University for Medical Sciences
- Omar El-Mukhtar Faculty of Medicine
- Alzaytunah University Faculty of Medicine
- Alasmariyah University Faculty of Medicine
- Sabha Faculty of Medicine
- Alkhums Faculty of Medicine
- Other
- **Level of Medical Education or Academic Year:**
- Preparatory Year
- First Year
- Second Year
- Third Year
- Fourth Year
- Fifth Year
- Internship
- **Have you had to relocate from your previous residence due to the civil war?**
- Yes
- No
- **Do you suffer from any ………… (tick all that apply)?**
- Health problems
- Psychological Problems
- Physical or learning disability
- **Do you have sufficient knowledge regarding the COVID-19 Pandemic?**
- Yes
- No
- **What is your source of information regarding the COVID-19 Virus? (tick all that apply)**
- WHO, CDC, UpToDate and other official sources?
- Local Government Statements
- Social Media groups and Pages
- Friends, Neighbors and Relatives
- Local and International Media Sources

***Medical Tele-Education***

- **What is your level of proficiency in using various electronic devices (Computers, Smart phones, etc…?)**
- Inadequate
- Acceptable
- Good
- Very Good
- Proficient
- **What type of internet service do you use primarily?**
- 4G
- 3G
- ADSL
- **How would you describe your internet service?**
- Bad
- Acceptable
- Good
- Very Good
- Excellent
- **Did you suspend your educational program (of your own volition) recently due to any of the following reasons?**
- No, I haven’t suspended my educational program
- Yes, I suspended my educational program due to the civil unrest / relocation from residence
- Yes, I suspended my educational program due to financial problems
- Yes, I suspended my educational program due to my social status and personal responsibilities
- Yes, I suspended my educational program due to other reasons
- **In general, do you suffer from any financial difficulties?**
- Yes
- No
- **Which of the following items do you personally own and utilize in your medical education?**

|  | Yes | No |
| --- | --- | --- |
| Personal Computer |  |  |
| A tablet or iPad |  |  |
| A smart Phone |  |  |

- **Does your phone device support any of the following technologies?**

|  | Yes | No |
| --- | --- | --- |
| Augmented Reality |  |  |
| High Definition Phone camera |  |  |
| Fourth Generation internet services (4G) |  |  |

- **Your educational program depends upon**

|  | **Yes** | **No** |
| --- | --- | --- |
| Lectures provided by the University |  |  |
| Courses provided by private education centers |  |  |
| Self-study utilizing various educational sources |  |  |

- **Have you used applications or information sources (Videos or images) that utilize Three-Dimensional Technology to understand concepts (such as anatomical dissection or steps for a particular surgical procedure) on your own personal electronic devices?**
- Yes
- No

**Medical courses and curriculum taught by your Faculty which you personally think that it would be difficult to teach through E-learning (whether theoretical or practical)**

----------------------------------------------------------------------------------------------------------------------------

- **What are your main uses for the internet?**

|  | Yes | NO |
| --- | --- | --- |
| Medical Education and E-learning |  |  |
| Social Media and E-mail |  |  |
| Tele-working |  |  |

***Education in your Medical Faculty***

- **Did your faculty suspend or postpone the educational program due to the COVID-19 Pandemic?**
- Yes
- No
- **Did your faculty suspend your clinical training program in response to COVID-19?**
- Yes
- No
- **Are you currently working in a Hospital?**
- Yes, as a physician / Intern under training
- Yes, as part of my educational program as a student in the clinical education stage / as a volunteer
- No, I do not currently work at the hospital
- I am still a student at the preclinical education stage, I neither work nor study at the hospital.
- **How are you spending your time during this period? (You may choose more than one answer)**
- My educational program was not disrupted
- Medical Education program is now online through online platforms
- I am engaged in self-development through a program not provided by my Faculty
- I am currently preparing for Medical License Exams
- Currently engaged in research or educational projects
- I participated in Volunteer Activities
- I help track down people who were in contact with confirmed COVID-19 patients
- I spend my time in resting and relaxing
- I look after an ill patient
- I feel unwell and have implemented self-isolation
- I am spending this time with my family
- I am trying to improve my physical fitness
- I watch TV
- I play video games
- I read non-medical books
- ecify :
- **How did COVID-19 Pandemic Affect your Career plan and Future Interests? (Select all that apply)**
- It has affected my career plan or future interests
- I became interested in Public Health
- I became interested in Infectious Medicine
- It has not affected my career plan or future interests

**What is your personal opinion regarding the following?**

Select the Answer that best describes the extent of your agreement with each statement

|  | Strongly Disagree | Disagree | Neutral | Agree | Strongly Agree | Not applicable |
| --- | --- | --- | --- | --- | --- | --- |
| It would have been better to help out in hospitals during the pandemic and continue my clinical training |  |  |  |  |  |  |
| I feel I limit my study potential due to COVID-19 |  |  |  |  |  |  |
| I am worried about losing chances to apply for specialty training due to COVID-19 |  |  |  |  |  |  |
| CoVID-19 has negatively affected my well-being |  |  |  |  |  |  |
| I am worried about being exposed to COVID-19 during my clinical practice or fulfillment of my duties at the hospital. |  |  |  |  |  |  |
| I am worried about being exposed to COVID-19 in my community |  |  |  |  |  |  |
| Responding to COVID will have almost no effect on my educational progress and Medical Career development |  |  |  |  |  |  |
| Responding to COVID-19 will have almost no effect on my qualification for training in specialties requiring a high degree of safe medical care provision |  |  |  |  |  |  |
| I admire the way my Medical Faculty is relaying information regarding impact of COVID-19 on our medical career progression, and gave us advice regarding what we should do (advice regarding continuation if our training program, Career development, etc…. |  |  |  |  |  |  |
| I am concerned regarding the effect this disruption may have on my medical career progression and training timeline, including specialty selection, evaluation exam participation, entering a specialty training program etc…. |  |  |  |  |  |  |

**Choose the response that best fits how you currently feel regarding the response of authorities to the COVID-19 pandemic**

|  | Strongly Disagree | Disagree | Neutral | Agree | Strongly Agree | Not applicable |
| --- | --- | --- | --- | --- | --- | --- |
| I am content with the response of our local government and authorities to the COVID-19 Pandemic |  |  |  |  |  |  |
| I am content with the response of health authorities my clinical specialty is associated with to the COVID-19 Pandemic |  |  |  |  |  |  |
| I am content with the response of official organizations representing medical students to the COVID-19 Pandemic (eg the Student Council) |  |  |  |  |  |  |

**The negative effect of the COVID-19 Pandemic on my Health Status:**

|  | Strongly Agree | Agree | Neutral | Disagree | Strongly Disagree |
| --- | --- | --- | --- | --- | --- |
| Physical Wellbeing (Physical activity and overall health) |  |  |  |  |  |
| Mental Wellbeing (stress and anxiety, mood disturbance) |  |  |  |  |  |
| Social Wellbeing (Social activities) |  |  |  |  |  |
| Intellectual Wellbeing (Ability to learn and understand) |  |  |  |  |  |
| Educational Wellbeing (Safe environment for working and learning) |  |  |  |  |  |

- **Which of the following concerns is troubling you at this time? (Tick all that apply)**
- My personal Health
- The Health of my family
- The overall health of the community
- My Financial Situation
- Someone else’s Financial Situation
- How my educational progress is affected by this pandemic
- **Are you more anxious now than you normally are during your studies?**
- Yes
- No
- **Do you practice social distances and stay at home as much as you can during this time?**
- Nearly all the time
- As much as possible
- No I don’t
- **Have any friends or relatives requested medical info or advice from you during the COVID-19 Pandemic?**
- Family
- Friends
- Others
- Have you used Social media to confer any information regarding the COVID-19 Pandemic?
- Facebook
- Twitter
- Instagram
- Other social media
- No, I don’t
- **How did COVID-19 affect your Elective or Internship?**
- I am still a pre-clinical level student
- I have completed my Elective training period
- Elective at my medical school was postponed
- My elective program was unaffected by the pandemic

**Over the last 2 weeks, how often have you been bothered by any of the following problems?**

| Feeling nervous, anxious or on edge? | | | | |
| --- | --- | --- | --- | --- |
| Not at all | Several Days | More than Half the days | | Nearly every day |
| Not being able to stop or control worrying? | | | | |
| Not at all | Several Days | More than Half the days | | Nearly every day |
| Worrying too much about different things? | | | | |
| Not at all | Several Days | More than Half the days | | Nearly every day |
| Trouble relaxing? | | | | |
| Not at all | Several Days | More than Half the days | | Nearly every day |
| Being so restless that it is hard to sit still? | | | | |
| Not at all | Several Days | More than Half the days | | Nearly every day |
| Becoming easily annoyed or irritable? | | | | |
| Not at all | Several Days | More than Half the days | | Nearly every day |
| Feeling afraid as if something awful might happen? | | | | |
| Not at all | Several Days | More than Half the days | | Nearly every day |
| Little interest or pleasure in doing things | | | | |
| Not at all | Several Days | More than Half the days | Nearly every day | |
| Feeling down, depressed or desperate | | | | |
| Not at all | Several Days | More than Half the days | Nearly every day | |

***Knowledge***

|  | True | False | I don’t know |
| --- | --- | --- | --- |
| E-Learning depends on a comprehensive digital electronic environment displaying educational curriculum through electronic networks |  |  |  |
| E-Learning is an interactive system that provides an opportunity for learning through Information and Telecommunication Technology |  |  |  |
| E-learning in the medical field is not considered less expensive than conventional learning |  |  |  |
| E-learning provides a digital multimedia content (written text, audio, video and images) |  |  |  |
| One of the benefits of E-learning with live content is that the scholar receives instant feedback from the instructor |  |  |  |
| E-learning is considered a type of tele-education |  |  |  |

***Attitude***

|  | Strongly Disagree | Disagree | Neutral | Agree | Strongly Agree |
| --- | --- | --- | --- | --- | --- |
| E-learning is applicable in Libya |  |  |  |  |  |
| E-Learning is a possible substitute for standard medical education |  |  |  |  |  |
| The E-Learning educational content that my University shall provide will satisfy all my educational requirements |  |  |  |  |  |
| Downloadable E-learning content is better than Live content |  |  |  |  |  |
| Adherence of students to work schedules for internet based educational content shall be similar to their adherence to direct learning content |  |  |  |  |  |
| An interactive electronic content is achievable (enabling you to ask questions and interact with the lecturer) similar to regular education |  |  |  |  |  |
| Most students in your faculty can use live online learning content |  |  |  |  |  |
| E-learning can be used for Clinical aspects of Medical Sciences |  |  |  |  |  |
| It is possible to give Private lessons through e-learning |  |  |  |  |  |
| It is possible to create educational content that covers the practical aspect of curriculum and courses you are currently studying |  |  |  |  |  |
| E-testing can replace traditional testing methods in medical faculties |  |  |  |  |  |
| Medical E-Learning is more convenient and flexible than conventional learning |  |  |  |  |  |
| The Quality of internet services in Libya can support E-learning Technology |  |  |  |  |  |
| It is possible to obtain material for medical educational through the internet |  |  |  |  |  |
| Interaction between students and lecturers is possible through E-learning technology |  |  |  |  |  |
| Civil War is prevents enterprises from establishing educational material on E-learning platforms |  |  |  |  |  |
| Libyan students have financial difficulties in gaining access to E-learning services (internet prices, procuring electronic devices) |  |  |  |  |  |
| Libyan Universities shall succeed in establishing E-learning programs for medical students |  |  |  |  |  |
| The veracity of Certificates attained through E-learning must be acknowledged |  |  |  |  |  |

***Practice***

|  | **Yes** | **No** |
| --- | --- | --- |
| Were you awarded certificates through online training courses related to the medical field |  |  |
| Did you participate in any online medical education program during this period organized by your faculty of medicine that is based on the medical educational curriculum at the faculty (Lectures or educational sessions using applications for online learning such as Zoom) |  |  |
| Did you use the internet to attend courses and information or understand concepts on various platforms (such as YouTube, khan academy etc.…?) |  |  |
| Do you download educational content related to your medical education in a periodic manner |  |  |
| Did you use online applications and platforms for the purpose of medical education (Coursera, Sololeran, edx, MOOCS) |  |  |
| Did you share medical educational material with your fellow medical students |  |  |
| Did you use the internet to study with a friend or a group of friends through online meetings |  |  |
| Did you use the internet to attend a course in Problem-based learning format |  |  |
| Do you utilize your personal computer in studying online |  |  |
| Do you use the internet regularly in your studies |  |  |
| Did you download electronic content instead of purchasing the paper format in order to save money |  |  |
| Did you purchase an electronic device in order to have access to E-learning opportunities |  |  |

- How would you describe your aptitude to participate in E-learning programs (lectures and training programs)?

| Ready now | **1** | **2** | **3** | **4** | **5** | Not ready at all |
| --- | --- | --- | --- | --- | --- | --- |
|  |  |  |  |  |  |  |

*Thank you for participating in this research study.*
