# Supplementary material for: Impact of the COVID-19 pandemic on medical education: Medical students’ knowledge, attitudes, and practices regarding electronic learning
Source: PLoS One. 2020 Nov 25;15(11):e0242905. doi: 10.1371/journal.pone.0242905 (PMC7688124; doi:10.1371/journal.pone.0242905)
Supplement: S2 File — (DOCX) [file pone.0242905.s003.docx]

**تاثير جائحة كورنا علي التعليم الطبي في ليبيا**

نرحب بمشاركتك في هذه المبادرة التي تهدف للرقي بالتعليم الطبي في ليبيا, لايجاد حلول للمشاكل التي يعاني منها طلاب الطب في بلادنا,نطلب منك تعبئة هذه البيانات ، والتي قد تستغرق 10 دقائق من وقتك ، هذا الاستبيان لا يحمل أي معلومات شخصية ، وسيتم استخدام البيانات التي يتم جمعها فقط لأغراض إحصائية وستبقى سرية ومجهولة المصدر ، ليتم استخدامها لأغراض إحصائية فقط ، نطلب منك أن تقدم إجاباتك بأمانة تامة لإظهار مدى تأثرك أثناء جائحة كورونا.

يعتبر إكمال هذا الاستبيان بمثابة موافقة من قبلكم للمشاركة في البحث. قد تكون نتائج البحث موضوع نشر في المستقبل

- **الجنس**
- ذكر
- أنثى
- **العمر بالسنوات**______________________
- الحالة الاجتماعية :
- متزوج
- أعزب

**هل عانيت من أي مشاكل مادية بسبب الأوضاع الحالية؟**

نعم

لا

**المدينة التي تعيش فيها**

__________________________________________

أنا طالب طب ______________

**ما هي كلية الطب التي تدرس بها ؟**

كلية الطب جامعة طرابلس

كلية الطب جامعة بنغازي

كلية الطب جامعة مصراتة

كلية الطب جامعة الزاوية

كلية الطب جامعة غريان

كلية الطب جامعة صبراتة

كلية الطب جامعة طبرق

الجامعة الليبية الدولية للعلوم الطبية

كلية الطب جامعة عمر المختار

كلية الطب جامعة الزيتونة

كلية الطب الجامعة الأسمرية

كلية الطب جامعة سبها

كلية الطب جامعة الخمس

كلية طب أخري

**السنة الدراسية أو المرحلة التعليمية**

إعداد الطب

سنة أولي

سنة ثانية

سنة ثالثة

سنة رابعة

سنة خامسة

طبيب امتياز

دراسات ما بعد التخرج / دراسات عليا

**هل أنت نازح من مكان سكنك بسبب الحرب ؟**

نعم

لا

**هل تعاني من ---------------؟**

| مشاكل صحية | نعم | لا |
| --- | --- | --- |
| مشاكل نفسية | نعم | لا |
| مشاكل جسدية أواعاقة | نعم | لا |

**هل لديك دراية كافية حول وباء الكورونا المستجد ؟**

نعم

لا

**ما هو مصدر معلوماتك حول فيروس كورونا المستجد؟**

| المواقع الرسمية مثل موقع WHO, CDC, UpToDate | نعم | لا |
| --- | --- | --- |
| المعلومات المحلية الرسمية الصادرة عن الجهات الحكومية | نعم | لا |
| صفحات ومجموعات التواصل الاجتماعي | نعم | لا |
| الأصدقاء, الجيران والأقارب | نعم | لا |
| وسائل الاعلام العالمية والمحلية | نعم | لا |

**التعليم الطبي عن بعد**

**ما مستوي قدراتك في استخدام الأجهزة الالكترونية المختلفة ؟ (الحاسوب, الهاتف المحمول الخ)؟**

ضعيف

مقبول

جيد

جيد جدا

ممتاز / محترف

**ما هي خدمة الانترنت التي تستعملها بشكل أساسي؟**

4G

3G

ADSL

**كيف تقيم خدمات الانترنت لديك ؟**

ضعيف

مقبول

جيد

جيد جدا

ممتاز

**هل قمت بايقاف قيدك أو برنامجك التعليمي (بقرار شخصي) مؤخرا لأي سبب من الأسباب الأتية ؟**

لا لم أوقف قيدي أو برنامجي التعليمي

نعم أوقفت قيدي / برنامجي التعليمي بسبب الوضع الأمني / النزوح

نعم أوقفت قيدي / برنامجي التعليمي بسبب الوضع المادي

نعم أوقفت قيدي / برنامجي التعليمي بسبب الوضع الاجتماعي والمسئوليات

نعم أوقفت قيدي / برنامجي التعليمي لأسباب أخري

**هل تواجه أي صعوبات مادية ومالية ؟**

نعم

لا

**أسئلة بخصوص الدراسة في كلية الطب**

**هل قامت كليتك بإيقاف أو تأجيل الدراسة بسبب فيروس كورونا ؟**

نعم

لا

**هل قامت كليتك بإيقاف برنامج التدريب السريري الخاص بك بسبب فيروس كورونا ؟**

نعم

لا

**هل تعمل في أحد المستشفيات حاليا ؟**

نعم أعمل بصفتي طبيب

نعم كجزء من البرنامج التعليمي كطالب في المرحلة السريرية / متطوع

لا أعمل حاليا في المستشفى

لا زلت طالبا/ة في المرحلة ما قبل لسريرية (لا أعمل ولا أدرس في المستشفى)

**كيف تقضي أوقاتك في هذه الفترة (يمكن اختيار أكثر من إجابة)**

| لم تتوقف دراستي أو تدريبي السريري | نعم | لا |
| --- | --- | --- |
| تحول التعليم إلي تعليم عن بعد عبر الانترنت بالتنسيق مع الكلية | نعم | لا |
| أقوم حاليا بتطوير نفسي والدراسة بشكل مستقل عن الكلية | نعم | لا |
| أدرس للتحضير لامتحانات المعادلة للشهادة الطبية | نعم | لا |
| أعمل في الأبحاث والبرامج العلمية | نعم | لا |
| أقضي أوقاتي في العمل التطوعي | نعم | لا |
| أساعد في البحث عن الأشخاص المخالطين للحالات المصابة بالفيروس | نعم | لا |
| اقضي وقتي في الراحة والاسترخاء | نعم | لا |
| أعتني بشخص مريض أو مقعد | نعم | لا |
| أنا لست بصحةجيدة وقمت بعزل نفسي | نعم | لا |
| أقضي الوقت في رعاية أطفالي وعائلتي | نعم | لا |
| أمارس نشاطات صحية وأقوم بتمارين رياضية أكثر من المعتاد | نعم | لا |
|  | نعم | لا |
| أشاهد التلفاز | نعم | لا |
| ألعاب الفيديو وألعاب الحاسوب | نعم | لا |
| قراءة الكتب (خارج مجال الطب) | نعم | لا |

|  | | | | | | نعم | لا |
| --- | --- | --- | --- | --- | --- | --- | --- |
| كيف أثر وباء كورونا علي قراراتك المهنية (مجال التخصص الذي تطمح إليه, خططك المستقبلية) ؟ (يمكن اختيار أكثر من إجابة | | | | | | | |
|  | ازدادت رغبتي في مجال الصحة العامة كتخصص مستقبلي |  |  | ازدادت رغبتي في مجالات أخري.  : | لم تؤثر في اهتماماتي وقراراتي المهنية | | |

| **نرجو الرد علي كافة الأسئلة باختيار أحد الردود الموجودة لتحديد مدي موافقتك لما ذكر (اختر أحد الإجابات فقط لكل سؤال)** | | | | | | |
| --- | --- | --- | --- | --- | --- | --- |
|  | **أعترض بشدة** | **أعترض** | **محايد** | **أوافق** | **أوافق بشدة** | **لا توجد إجابة** |
| كنت أفضل لو أنني تمكنت من المساعدة في المستشفيات في هذا الوقت, واستكمال دراستي العملية. |  |  |  |  |  |  |
| أشعر بأنني لا أسخر طاقاتي كما يجب في هذا الوقت |  |  |  |  |  |  |
| لن أتضايق إذا ساعدت في رعاية المرضي في المستشفيات في هذا الوقت |  |  |  |  |  |  |
| أنا قلق بشأن تأثير كورونا علي فرصتي لنيل التخصص الذي أطمح إليه  (إذا كنت حاليا تعمل في تخصص معين اختر لا يوجد إجابة) |  |  |  |  |  |  |
| وباء كورونا أثر علي صحتي بشكل سلبي |  |  |  |  |  |  |
| أنا قلق بشأن احتمال تلقي عدوي فيروس كورونا أثناء تأدية مهامي في المستشفى |  |  |  |  |  |  |
| أنا قلق بشأن احتمال تلقي عدوي فيروس كورونا من المجتمع |  |  |  |  |  |  |
| المساعدة في مواجهة فيروس كورونا سيكون لها تأثير بسيط في قدرتي علي التعلم وتطوير مهاراتي |  |  |  |  |  |  |
| المساعدة في مواجهة فيروس كورونا سيكون لها تأثير بسيط في قدرتي علي دخول تخصص يحتاج معرفة ومهارات في توفير رعاية صحية أمنة عالية المستوي |  |  |  |  |  |  |
| أنا راض عن الطريقة التي قامت مؤسستنا التعليمية بإيصال المعلومة حول تأثير وباء كورونا علينا كطلاب والخطوات القادمة التي يجب علي القيام بها كطالب (استكمال باقي برنامجي التدريبي, التطور المهني في حياتي, إلخ.........) |  |  |  |  |  |  |
| أنا قلق بشأن تأثير هذا الفاصل الزمني علي تقدمي العلمي والجدول الزمني للتدريب الطبي, ويشمل اختيار التخصص, اتباع جدول زمني محدد, استكمال اختبارات معادلة الشهادة الطبية في موعدها, والدخول في برنامج تدريب تخصصي في موعده |  |  |  |  |  |  |

| **نرجو الرد علي كافة الأسئلة باختيار أحد الردود الموجودة لتحديد مدي موافقتك لما ذكر (اختر أحد الإجابات فقط لكل سؤال)** | | | | | | |
| --- | --- | --- | --- | --- | --- | --- |
|  | **أعترض بشدة** | **أعترض** | **محايد** | **أوافق** | **أوافق بشدة** | **لا توجد إجابة** |
| أنا راض عن استجابة الحكومة والسلطات المحلية في المدينة التي أسكن فيها لوباء كورونا |  |  |  |  |  |  |
| أنا راض عن استجابة المنظمات الرسمية التي يتبعها التخصص العملي الذي أعمل فيه لوباء كورونا |  |  |  |  |  |  |
| أنا راض عن استجابة المنظمات الرسمية الخاصة بطلبة الطب لوباء كورونا (منظمات خاصة بطلبة الطب, اتحادات خاصي بمن يدرس التخصص) |  |  |  |  |  |  |

|  |
| --- |

| **وباء كورونا أثر سلبا علي النواحي الاتية من صحتي** | | | | | | |
| --- | --- | --- | --- | --- | --- | --- |
| الصحة الجسدية (تشمل الرياضة, الغذاء, تعاملك مع المرض) | **أعترض بشدة** | **أعترض** | **محايد** | **أوافق** | **أوافق بشدة** | **لا توجد إجابة** |
| الصحة النفسية (المزاج, القلق, التوتر, الصحة العاطفية) |  |  |  |  |  |  |
| الصحة الاجتماعية (احساسك بالاهتمام, المساواة) |  |  |  |  |  |  |
| الصحة العقلية (القدرة علي التعلم وتحقيق الأهداف العلمية) |  |  |  |  |  |  |
| الصحة العملية (القدرة علي التعلم والعمل في بيئة ءامنة) |  |  |  |  |  |  |

**أي من الاتي تعتبر مخاوف أو مصدر للقلق لك في هذا الوقت ( يمكن اختيار أكثر من إجابة)**

| صحتي وسلامتي الشخصية | نعم | لا |
| --- | --- | --- |
| صحة وسلامة عائلتي | نعم | لا |
| صحة وسلام المجتمع | نعم | لا |
| وضعي المادي | نعم | لا |
| الوضع المادي لأشخاص أهتم لأمرهم | نعم | لا |
| تأثير الوباء علي قدرتي في استكمال تعليمي | نعم | لا |

| **هل تعتبر مستوي القلق لديك أعلي من المعتاد خلال سنوات الدراسة أو دراسة التخصص** | | | | | | | | | | | | | | | |
| --- | --- | --- | --- | --- | --- | --- | --- | --- | --- | --- | --- | --- | --- | --- | --- |
| نعم | | | | | لا | | | | | أفضل عدم الاجابة | | | | | |
| **هل تحافظ علي مبدأ المسافة الأمنة (البقاء في المنزل قدر المستطاع لمنع انتشار وباء كورونا) (اختر إجابة واحدة)** | | | | | | | | | | | | | | | |
| بشكل شبه كامل | | | | | قدر المستطاع | | | | | لا أبدا | | | | | |
| **هل يطلب منك أفراد أسرتك أو أصدقائك تقديم النصائح أو المعلومات الطبية فيما يخص فيروس كورونا (يمكن اختيار أكثر من إجابة)** | | | | | | | | | | | | | | | |
| (أفراد الأسرة) نعم لا | | | | | (الأصدقاء نعم لا | | | | | (أشخاص ءاخرون) | | | نعم لا | | |
| **هل تستخدم وسائل التواصل الاجتماعي لإيصال المعلومات للأخرين حول وباء كورونا ؟ (يمكن اختيار أكثر من إجابة)** | | | | | | | | | | | | | | | |
| نعم (تويتر) | | نعم (الفيسبوك) | | | | نعمل (انستاجرام) | | | نعم وسائل تواصل أخري | | | | | | لا |
| **كيف أثر وباء كورونا علي برنامج الامتياز لديك؟** | | | | | | | | | | | | | | | |
|  | لا زلت في مرحلة الدراسة الجامعية قبل سنة الامتياز | |  | أكملت الامتياز | | |  | تم تأجيل سنة الامتياز | | |  | لم يتأثر برنامج الامتياز | |  | |

**هل تعمل حاليا في مجال بعيد عن مجال الطب لتقوم بتوفير نفقات تعليمك أو تدريبك ؟**

نعم

لا

| **كم عانيت من المشاكل التالية خلال الأسبوعين الماضيين (ضع علامة √ أمام الإجابة الأنسب)** | **أبدا** | **بعض الأيام** | **أغلب الأيام** | **كل الأيام تقريبا** |
| --- | --- | --- | --- | --- |
| الشعور بالغضب أو القلق أو الانفعال الشديد |  |  |  |  |
| عدم القدرة على إنهاء القلق أو التحكّم فيه |  |  |  |  |
| القلق المفرط على أشياء مختلفة |  |  |  |  |
| الصعوبة في الاسترخاء |  |  |  |  |
| شدة الاضطراب لدرجة صعوبة البقاء ساكنا |  |  |  |  |
| السرعة في الانزعاج أو الانفعال |  |  |  |  |
| الشّعور بالخوف كما لو أن شيئا سيئا قد يحدث |  |  |  |  |
| قلة الاهتمام أو عدم إيجاد سعادة عند القيام بإنجاز أي شئ |  |  |  |  |
| الإحساس بالاكتئاب أو اليأس أو الإحباط |  |  |  |  |

| يعتمد التعليم الالكتروني علي بيئة الكترونية رقمية متكاملة تعرض المقررات الدراسية عبر الشبكات الالكترونية | نعم | لا | لا أدري |
| --- | --- | --- | --- |
| التعليم الالكتروني هو نظام تفاعلي يقدم فرصة للتعلم باستخدام تقنية الاتصالات والمعلومات | نعم | لا | لا أدري |
| لا يعتبر التعليم الطبي الالكتروني قليل التكلفة مقارنة بالتعليم المباشر | نعم | لا | لا أدري |
| يقدم التعليم الالكتروني محتوي رقمي متعدد الوسائط (نصوص مكتوبة أومنطوقة, فيدوات وصور) | نعم | لا | لا أدري |
| من مميزات التعليم الالكتروني ذو المحتوي المباشر حصول المتعلم علي تأدية | نعم | لا | لا أدري |
| يعتبر التعليم الالكتروني أحد أنواع التعليم عن بعد | نعم | لا | لا أدري |

**التعليم الطبي عن بعد**

**التعليم الالكتروني قابل للتطبيق في ليبيا**

أوافق بشدة

أوافق

محايد

لا أوافق

لا أوافق بشدة

**التعليم الالكتروني يمكن أن يصبح بديلا للتعليم الطبي المباشر**

أوافق بشدة

أوافق

محايد

لا أوافق

لا أوافق بشدة

**التعليم الالكتروني سهل التطبيق في ليبيا**

أوافق بشدة

أوافق

محايد

لا أوافق

لا أوافق بشدة

**المحتوي التعليمي الالكتروني الذي ستقدمه الجامعة سيلبي كافة احتياجاتك التعليمية**

أوافق بشدة

أوافق

محايد

لا أوافق

لا أوافق بشدة

**المحتوي الالكتروني القابل للتحميل أفضل من المحتوي المباشر**

أوافق بشدة

أوافق

محايد

لا أوافق

لا أوافق بشدة

**التزام الطلبة بجدول مواعيد المادة التعليمية المقدمة علي الانترنت سيكون مشابه لالتزامهم بالمادة المقدمة بشكل مباشر**

أوافق بشدة

أوافق

محايد

لا أوافق

لا أوافق بشدة

**من الممكن صناعة محتوي الكتروني تفاعلي (بحيث يمكنك الاستفسار والتفاعل مع الأستاذ المحاضر) كما هو مشاهد في التعليم المباشر**

أوافق بشدة

أوافق

محايد

لا أوافق

لا أوافق بشدة

**أغلب الطلبة من أصدقائك وكليتك لديهم القدرة والامكانيات اللازمة لاستخدام التعليم المباشر**

أوافق بشدة

أوافق

محايد

لا أوافق

لا أوافق بشدة

**من الممكن استخدام التعليم الالكتروني لتعليم الجانب السريري (الكلينيكي) من العلوم الطبية**

أوافق بشدة

أوافق

محايد

لا أوافق

لا أوافق بشدة

**من الممكن إعطاء دروس خصوصية عن طريق التعليم الالكتروني لكي يتمكن الطالب من فهم ما هو معقد**

أوافق بشدة

أوافق

محايد

لا أوافق

لا أوافق بشدة

**من الممكن صنع محتوي يغطي الجانب العملي من الدورات والمواد التي تدرسها حاليا**

أوافق بشدة

أوافق

محايد

لا أوافق

لا أوافق بشدة

**من الممكن إنشاء اختبارات الكترونية عن بعد تغني عن الاختبارات التقليدية**

أوافق بشدة

أوافق

محايد

لا أوافق

لا أوافق بشدة

**التعليم الطبي الالكتروني أكثر مرونة من التعليم الاعتيادي**

أوافق بشدة

أوافق

محايد

لا أوافق

لا أوافق بشدة

**جودة خدمات الانترنت في ليبيا تلبي احتياجات استخدام الطلبة لتقنية التعليم الالكتروني**

أوافق بشدة

أوافق

محايد

لا أوافق

لا أوافق بشدة

**من الممكن الحصول علي المواد التعليمية الطبية عن طريق الانترنت**

أوافق بشدة

أوافق

محايد

لا أوافق

لا أوافق بشدة

**التفاعل بين الطلبة والمحاضرين ممكن من خلال تقنية التعليم الالكاروني**

أوافق بشدة

أوافق

محايد

لا أوافق

لا أوافق بشدة

**الحرب صعبت علي الجهات المعنية اعتماد محتي علمي علي منصات التعليم الالكتروني**

أوافق بشدة

أوافق

محايد

لا أوافق

لا أوافق بشدة

**الطلاب الليبيين سيصعب عليهم استخدام التعليم الالكتروني لأسباب مادية**

أوافق بشدة

أوافق

محايد

لا أوافق

لا أوافق بشدة

**الجامعات الليبية ستنجح في تطبيق التعليم الالكتروني**

أوافق بشدة

أوافق

محايد

لا أوافق

لا أوافق بشدة

يجب الاعتراف بمصداقية الشهادات المتحصل عليها عن طريق التعليم الالكتروني

أوافق بشدة

أوافق

محايد

لا أوافق

لا أوافق بشدة

**الممارسة**

| هل حصلت علي شهادات من دورات تدريبية الكترونية في المجال الطبي | نعم | لا |
| --- | --- | --- |
| هل شاركت في برنامج تعليم طبي عن بعد نظمته الكلية التي تدرس بها يتبع المنهج الدراسي بالكلية | نعم | لا |
| هل كنت تستخدم الانترنت للحصول علي معلومات ومحاضرات لفهم مناهج طبية | نعم | لا |
| هل تقوم بتحميل محتوي تعليمي من خلال الانترنت يتعلق بدراستك الطبية | نعم | لا |
| هل تستخدم تطبيقات الكترونية ومواقع لمنصات تعليمية لغرض التعليم الطبي | نعم | لا |
| هل قمت بمشاركة محتوي تعليمي طبي مع زملائك في الكلية | نعم | لا |
| هل قمت بالدراسة الطبية مع زملاء عبر الانترنت | نعم | لا |
| هل قمت باستخدام الانترنت لحضور محاضرات مصممة بطريقة problem based learning | نعم | لا |
| هل تستخدم جهاز الحاسوب الخاص بك للدراسة باستخدام الانترنت | نعم | لا |
| هل تستخدم الانترنت بشكل رسمي في دراستك | نعم | لا |
| هل قمت بتحميل محتوي الكتروني بدلا من شراء نسخة ورقية لغرض توفير المال | نعم | لا |
| هل قمت بشراء جهاز الكتروني لكي تحصل علي فرص تعليم الكتروني | نعم | لا |

**كيف تصف استعدادك للمشاركة في برامج تعليمية الكترونية**

غير مستعد بتاتا

غير مستعد حاليا

مستعد قليلا

مستعد بشكل جيد

مستعد حالا

**أي مما يلي تستخدم في دراستك الطبية (يمكناختيار أكثر من إجابة)**

الحاسوب الشخصي

جهازلوحي

هاتف ذكي

**هل يدعم هاتفك أي من التقنيات الاتية**

| تقنية الواقع الافتراضي المعزز | نعم | لا |
| --- | --- | --- |
| تقنية واي فاي | نعم | لا |
| كاميرا عالية الوضوح | نعم | لا |
| تقنية اتصالات الجيل الرابع | نعم | لا |

**يعتمد برنامجك الدراسي علي:**

| المحاضرات المقدمة من قبل الكلية الطبية | نعم | لا |
| --- | --- | --- |
| دورات تقوية مقدمة من مراكز التعليم الخاص | نعم | لا |
| أقوم بالدراسة بمفردي بالاستعانة بمواد تعليمية مختلفة | نعم | لا |

**هل قمت باستخدام تطبيقات أو مصادر تستخدم تقنية ثلاثية الأبعاد علي جهازك لفهم مواد طبية**

نعم

لا

**ما هي المادة الطبية (نظرية أو عملية) التي تعتقد أنه من الصعب استخدام تقنية التعليم الالكتروني لتعليمها**

_______________________________________________________________

**ما هي استخداماتك الأساسية للانترنت ؟**

| الدراسة الطبية والتعليم الالكتروني | نعم | لا |
| --- | --- | --- |
| وسائل التواصل الاجتماعي والبريد الالكتروني | نعم | لا |
| العمل عن بعد | نعم | لا |
| المطالعة والتصفح | نعم | لا |
