# Supplementary material for: Impact of the COVID-19 pandemic on medical education: Medical students’ knowledge, attitudes, and practices regarding electronic learning
Source: PLoS One. 2020 Nov 25;15(11):e0242905. doi: 10.1371/journal.pone.0242905 (PMC7688124; doi:10.1371/journal.pone.0242905)
Supplement: Attachment — Submitted filename: PONE-D-20-22931_Review lib med ed.docx [file pone.0242905.s005.docx]

**ID:** PONE-D-20-22931

**Title:** The Impact of the COVID-19 Pandemic on Medical Education: Medical Students’ Knowledge, Attitudes, and Practices Regarding Electronic Learning

**Publication Criteria:**

1. The study presents the results of original research.
   a. Yes
2. Results reported have not been published elsewhere.
   a. Yes
3. Experiments, statistics, and other analyses are performed to a high technical standard and are described in sufficient detail.
   a. Not entirely
4. Conclusions are presented in an appropriate fashion and are supported by the data.
   a. No
5. The article is presented in an intelligible fashion and is written in standard English.
   a. Yes
6. The research meets all applicable standards for the ethics of experimentation and research integrity.
   a. Yes
7. The article adheres to appropriate reporting guidelines and community standards for data availability.
   a. No
   b. Comments: The authors reported that “the data underlying the results presented in the study are available from the author upon request.” Publication guidelines for this journal specifically note that “stating ‘data available on request from the author’ is not sufficient. If your data are only available upon request, select ‘No’ for the first question and explain your exceptional situation in the text box.” The authors wrote that “some restrictions will apply” but did not explain what these restrictions, why these restrictions are appropriate, and how their situation is an extenuating circumstance re: data availability.

**Reviewer Comments:**

I appreciate the timing and value of this study on medical students’ perceptions of online learning in Libya in the context of the ongoing COVID-19 pandemic and the civil war. It is a remarkable testimonial to the commitment and resilience of the students and the faculty that such serious medical education is continuing. Also, impressive that the authors were able to collect and analyze such an extensive set of data under these circumstances. As the authors note in their introduction, the pandemic has disrupted medical education, and it is important for medical educators around the world to adapt to these extenuating circumstances.

There are a couple strengths of the study:

1. The study included more than 10 different medical schools in Libya, a particularly challenging but not entirely unique context for medical education, and they collected data from students across all different class years with a high survey response rate.
2. The survey used in the study included measures across many different domains, allowing extensive triangulation about Libyan medical students’ circumstances and knowledge, attitudes, and practices related to online learning.

Overall, I appreciate the findings the authors reported in this study and believe their data provide valuable information about the challenges of adapting medical education in the midst of the ongoing pandemic. However, I would strongly recommend a number of aspects of the analysis and presentation be substantially revised for this study to be considered for publication. These are listed below, roughly in order as they appear in the publication. The most serious issues are in bold. See item #12 in particular as a restructuring of the paper that is needed, and #8 in particular as something that I would see as a rather large but essential change in the analysis.

1. The authors mention that the survey was distributed to “more than 13 medical schools in Libya” (line 104), but they do not state the exact number in their manuscript. They should report the exact number of medical schools included in the dataset analyzed for the study.
2. There is a very large difference in the number of respondents from the different schools, with three schools having large fraction of the total. It would be useful to break the analysis down between the three large and many small institutions, or at least large and small number of respondents, as I would expect there could be substantial differences between the two groups
3. The authors mention that their analysis excluded questionnaires that were either incomplete or contained missing data (lines 106-107). However, they do not report in in the “Results” section how many questionnaires were excluded based on these criteria. The authors should report this number.
4. The authors provide only an estimated response rate (line 165). The authors should abide by common reporting practices and indicate the exact response rate.
5. **71.4% of respondents were female and only 28.6% male (line 167). The authors do not provide an explanation or discussion of this dramatic skew towards female participants and whether these percentages are representative of the female/male breakdown of medical students in Libya. Discussion of this skew is particularly important to include given that Table 1 shows that several important characteristics of the study population – for example having financial issues, currently displaced/relocated, and physical or learning disability – are significantly different between females and males in their study population.**
6. For Figure 1, rather than a pie chart, the authors should report their findings as a table, as per typical reporting standards for the type of data they include in this figure.
7. **They list the educational experiences on questionnaire as “Lectures provided by the University. Courses provided by private education centers / courses. Self-study utilizing various educational sources”. While these labels are apparently well understood by the respondents, there should be brief explanations as to what each of these would entail for benefit of international readership. In particular, the label “lecture”, can encompass some quite ineffective pedagogical practices, as well as some effective ones depending on the context and who is using it. I am assuming here they mean only the basic meaning of simple transmission of information via an instructor talking, with no interaction with students or between students. If any of these methods involve more than just basic information transfer and did involve various types of interaction and formative assessment, that needs to be examined in more detail in the analysis. It would be quite misleading to lump together the perceptions of students that experienced such different educational experiences.**
8. **On line 268, they say that only 27% of the respondents had experienced e-learning. It makes no sense to lump together the attitudes and perceptions of e-learning of the ¾ of the students that have never experienced e-learning with this ¼ that have. In fact, I would argue it is not very important what that ¾ thinks, and it would be much more meaningful to only look at the results of all the e/online learning questions for those ¼ of the students that have actually experienced it.**
9. **The authors make the conclusion in the “Discussion” section that their study findings indicate adequate knowledge, attitudes, and practice levels to support implementation of online learning in Libya (lines 453-454). I disagree with this conclusion. The authors reported in their “Results” section that 73.6% of respondents believed the quality of the local internet was not sufficient to facilitate online learning platforms (lines 273-274), 66.5% believed conflicts in Libya could pose challenges for online learning (lines 274-275), 78.3% found it difficult to participate in online learning because of financial costs (lines 275-276), and only 20.2% believed that Libyan medical schools could implement online throughout the pandemic (lines 276-277). As the authors describe in their “Introduction” section, the goal of this study was to assess the feasibility of online learning for medical students (lines 96-97). Their reported findings suggest that there will be significant barriers to implementing online learning for Libyan medical students based on the findings described above. The authors’ “Discussion” section does not acknowledge these barriers. I would strongly recommend the authors revise their “Discussion” section to better reflect the findings reported in their “Results” section and provide a discussion of potential strategies that will need to be implemented in order for online learning to be feasible.**
10. There is lots of discussion at end about possible things to do, but not based on any of their data, just repeating material published and well known elsewhere, without any particular justification that it would work in this context. This does not seem to add anything worthwhile to the manuscript.
11. **The “Discussion” section does not include any direct discussion of limitations of this study; the authors only make suggests for future research (lines 448-452). The authors should describe specific limitations of their study and findings such that readers can interpret their findings in context. As part of this, they should also discuss what aspects of their findings could be generalized and to what contexts. In particular, what aspects would likely be relevant to many other countries struggling to provide medical education in the presence of covid and other major civil disruptions, and what aspects are more likely relevant only to the Libya context?**
12. **A more general comment concerns the focus of the manuscript. It presents a large amount of information but there is little attention as to which is important and/or relevant to covid and their research questions, and which is not. The entire paper needs to be more focused, with better defined research questions and results, rather than such a long tabulation of numbers, some of which are significant in terms of educational methods and policy decisions and many are not.**
