# Supplementary material for: Impact of the COVID-19 pandemic on medical education: Medical students’ knowledge, attitudes, and practices regarding electronic learning
Source: PLoS One. 2020 Nov 25;15(11):e0242905. doi: 10.1371/journal.pone.0242905 (PMC7688124; doi:10.1371/journal.pone.0242905)
Supplement: Attachment — Submitted filename: Author responses to reviewers September 2020.docx [file pone.0242905.s006.docx]

Dear Dr. Yuka Kotozaki

Academic Editor

PLOS ONE

Thank you very much for this great opportunity to revise our manuscript. We really appreciate your time and efforts to revise our manuscript.

I have tried to respond to reviewers and editorial comments using Red color for my response, kindly check with the track change version to demonstrate the required changes that were done.

You will find the following fines:

Manuscript - V2 PLoS ONE Track Change

This version contain the required changes that was done in response to reviewers comments.

Manuscript_-_V2_PLoS_ONE_-_Language Editing

This version contains many significant changes that were done in order to organize and edit the language and organize some parts of the paper to be able to reach the highest possible quality standard of PLOS One especially after reviewers’ comments about editing some parts and avoid repeating of information, therefore the manuscript has been edited heavily to improve clarity, word choice, and sentence structure. Please check carefully if possible.

Manuscript_-_V2_PLoS_ONE_-_Clean Version

This is the final version that is clean without track changes and contain the latest edit after responding to reviewers’ comments.

Journal Requirements:

When submitting your revision, we need you to address these additional requirements.

1. Please ensure that your manuscript meets PLOS ONE's style requirements, including those for file naming. The PLOS ONE style templates can be found at

<https://journals.plos.org/plosone/s/file?id=wjVg/PLOSOne_formatting_sample_main_body.pdf> and

<https://journals.plos.org/plosone/s/file?id=ba62/PLOSOne_formatting_sample_title_authors_affiliations.pdf>

 Thank you very much for your comment.

I have revised the manuscript and edit it according to journal guideline. Please check with the final clean version if possible.

2.We note that you have indicated that data from this study are available upon request. PLOS only allows data to be available upon request if there are legal or ethical restrictions on sharing data publicly. For information on unacceptable data access restrictions, please see <http://journals.plos.org/plosone/s/data-availability#loc-unacceptable-data-access-restrictions>.

 In your revised cover letter, please address the following prompts:

a) If there are ethical or legal restrictions on sharing a de-identified data set, please explain them in detail (e.g., data contain potentially identifying or sensitive patient information) and who has imposed them (e.g., an ethics committee). Please also provide contact information for a data access committee, ethics committee, or other institutional body to which data requests may be sent.

b) If there are no restrictions, please upload the minimal anonymized data set necessary to replicate your study findings as either Supporting Information files or to a stable, public repository and provide us with the relevant URLs, DOIs, or accession numbers. Please see <http://www.bmj.com/content/340/bmj.c181.long> for guidelines on how to de-identify and prepare clinical data for publication. For a list of acceptable repositories, please see <http://journals.plos.org/plosone/s/data-availability#loc-recommended-repositories>.

We will update your Data Availability statement on your behalf to reflect the information you provide.

 Thank you very much.

I have read the data access availability regulations of PLOS One.

Therefore, I have uploaded all the raw data of the study as “Data in Supporting Information files”.

You can find this in our file of the study, and if you could if possible, edit this to be in accordance with the new addition and editing, that would be greatly appreciated. The data are in SPSS, however if you want us to change it to excel or other types, please let me know if possible although SPSS might be appropriate and easier to be used.

Please ensure that PLOS One will have the copyright of the manuscript and related data, so please if you could make a note that the data must be cited within the paper if used by other researchers.

3. Your ethics statement must appear in the Methods section of your manuscript. If your ethics statement is written in any section besides the Methods, please move it to the Methods section and delete it from any other section. Please also ensure that your ethics statement is included in your manuscript, as the ethics section of your online submission will not be published alongside your manuscript.

 Thank you very much.

I have moved it into the method section of the manuscript as instructed.

4. Please include captions for your Supporting Information files at the end of your manuscript, and update any in-text citations to match accordingly. Please see our Supporting Information guidelines for more information: http://journals.plos.org/plosone/s/supporting-information.

I have edited the supporting information files as follows

**Figure Caption**

**Fig 1. Distribution of Medical Students among Medical Schools Included in the Study (n = 3348).**

**Supplementary Files**

**Supplementary Table 1. Distribution of Medical Students according to Medical Schools**

**Supplementary File 1 English Version of the Questionnaire**

**Supplementary File 2 Arabic Version of the Questionnaire**

Reviewers' comments:

Reviewer's Responses to Questions

**Comments to the Author**

1. Is the manuscript technically sound, and do the data support the conclusions?

The manuscript must describe a technically sound piece of scientific research with data that supports the conclusions. Experiments must have been conducted rigorously, with appropriate controls, replication, and sample sizes. The conclusions must be drawn appropriately based on the data presented.

Reviewer #1: Yes

Reviewer #2: No

2. Has the statistical analysis been performed appropriately and rigorously?

Reviewer #1: Yes

Reviewer #2: No

3. Have the authors made all data underlying the findings in their manuscript fully available?

The [PLOS Data policy](http://www.plosone.org/static/policies.action#sharing) requires authors to make all data underlying the findings described in their manuscript fully available without restriction, with rare exception (please refer to the Data Availability Statement in the manuscript PDF file). The data should be provided as part of the manuscript or its supporting information, or deposited to a public repository. For example, in addition to summary statistics, the data points behind means, medians and variance measures should be available. If there are restrictions on publicly sharing data—e.g. participant privacy or use of data from a third party—those must be specified.

Reviewer #1: Yes

Reviewer #2: No

4. Is the manuscript presented in an intelligible fashion and written in standard English?

PLOS ONE does not copyedit accepted manuscripts, so the language in submitted articles must be clear, correct, and unambiguous. Any typographical or grammatical errors should be corrected at revision, so please note any specific errors here.

Reviewer #1: Yes

Reviewer #2: Yes

Thank you very much for your answers, I will try to address your kind comments accordingly.

5. Review Comments to the Author

Reviewer #1: This review is actually three independent reviews done in the context of a Masters degree program.

Thank you very much for your kind comment, I will try to address all comments accordingly.

Reviewer: B
Summary:
The Impact of the COVID-19 Pandemic on Medical Education: Medical Students’ Knowledge, Attitudes, and Practices Regarding Electronic Learning is a manuscript written to address the issue of majority suspended medical education in Libya during the time of the COVID-19 pandemic. The authors address not only the students’ opinions and attitudes related to whether e-learning is possible and/or plausible while at the same time being an effective way to learn what the students would otherwise be learning within the hospital setting.
The study addresses the feasibility of e-learning however, that is only achieved by looking at access to electronics and internet connection, though that is an important point made. While live and recorded lectures can be easily done virtually and as effectively, this cannot be achieved without internet. An interesting discussion point that I would have liked embellished upon was that of the civil war’s impact on these students and their financial and personal stability, as this is a large part of Libya today. In the introduction to the study, the notion of feasibility of online learning was noted several times but nothing was really gained from this repetition.

Thank you very much for your kind comment.

I really appreciate your kind feedback and really helpful for us.

We appreciate the time and efforts to revise the manuscript to help us to improve our manuscript.

Regarding the repeating, we tried to focus more on the online learning, though we tried also as you mentioned to measure its feasibility in Libya as most of the medical schools are closed for lectures until this moment.

Major Points:
• The idea that virtual clinical experience is in any way a substitute is an assumption and while the pandemic is ongoing, the idea of a safe environment may not exist. I would prefer the wording to be more ambiguous, such as making the environment as safe as possible. (conclusion) The authors need to be clear that suggesting there will ever be a healthcare setting that is “safe from” COVID seems far-fetched.

I totally agree with you about the virtual experience although we referenced some examples that were able to use it, however it may need more time and other studies to address it in further details.

Also, I agree with you about the safe environment issue, I have edited the conclusion paragraph that contain this sentence as follow:

" Valid solutions are needed to reduce this disruption, and such measures may take the form of online training and virtual clinical experience, followed by hands-on experience in a safe environment, although the latter may take time considering the continued spread of COVID-19”

Although as stated in this section of the manuscript that these are suggestion based from the authors for future implementation and they will be recommendations for any efforts that may support these initiatives which we hope so.

• Are medical students primarily female in Libya? If not very interesting that there was such a discrepancy between males and females that decided to respond. This variance should be addressed as the difference in number was significant and the authors during the conclusion made several statements related to the variances between the genders.

Yes, the medical students in Libya are primary female, although there is no official figures and numbers, but based on the local experience, most of the medical students are female, adding to this, they are more active and they usually participate more than male in activities and research projects. Even though we found such discrepancy between male and female medical students. We tried to make this as a note in the statements as there was some associations and discrepancy between male and female in the study.

• Suspending clinical rotations was likely necessary with the first large wave and unknown nature of the disease but should be constantly re-assessed as it was determined this would not be a virus that is seasonal or likely to truly disappear any time in the foreseeable future. (75) When follow-up to this is done, information from the medical schools regarding their plans to re-assess the suspensions would be appreciated.

I agree with you, however unfortunately most of the schools did not resumed the clinical rotations except for interns in a very narrow and specific ways in orders for them to complete their graduation, while other clinical years are still suspended until the current moments in most of the universities and this may have negative effects on the students which we have addressed through the discussion about providing somehow virtual clinical experience for medical students. Therefore, we hope that there would be a future plans to assess this suspensions and whether or not there would be new strategies aiming to provide interventions or solution to these issues.

• The question continues to be posed if online learning is feasible for medical students. (96) If students have adequate connection and are required to stay home, how lectures (live or downloadable) would not be feasible and at least provide some semblance of education.

You are right, however not all students are able to use the online learning, there are still many other obstacles although our study tried to provide some answers and evaluation of the current status, however other challenges such as electricity black out issues, the internet issues, and some students are living outside of the main cities which may not have adequate internet supply during the lockdown of COVID-19.

Another major issues, that we mentioned in this sentence that you mentioned is that some departments have started to provide online lectures, however this was based on few departments not all of them, and not part of the official plan that should be done for the universities for online learning, because only few departments and lecturers have done that, which means that we need a strategic plan by the universities and authorities to turn on all lectures into online with specific schedule and programs.

• Lines 400-415 mention telemedicine and interactive online cases which are proposed methods but would benefit the conclusion/suggestions with more details and description.

We tried to provide some details and definitions of these methods, however there are still many to do in future and still now there was no studies that measured this as proposed method or alternative for the usual clinical training. These were discussed in the discussion part in fewer details, however we tried to summarize our findings in the conclusion. Also, we mentioned them in some details in the conclusion section as follows:

" We recommend adapting interactive online learning lectures by using highly sophisticated technologies along with virtual clinical experience to combine clinical scenarios with similar bedside teaching based on discussions of medical cases. Such measures would help students adapt to this way of medical teaching.”

Also, " The COVID-19 pandemic is ongoing and will continue to disrupt medical education and training. COVID-19 has overloaded the healthcare system and affected the ability of healthcare providers to provide adequate healthcare services. As we face a second wave of this outbreak, we must undertake several measures and make changes, so as to minimize the impact on medical education and the progression of training. Valid solutions are needed to reduce this disruption, and such measures may take the form of online training and virtual clinical experience, followed by hands-on experience in a safe environment."

• Line 453 says their findings show adequate knowledge of e-learning, though the findings seem more indicative of the students’ knowledge that is exists, not in so much as they are familiar with actually using it or would be able to.

Thank you for your comment, we tried to assess the knowledge and practice of e-learning, which our results are indicative with their ability to use it and support implementing this program. However, as most of the medical school did not provided official program for it, then we may not be able to know if all students are familiar or actually using it. However, further studies are needed.

Minor Points:
• Well done noting the survey was provided in Arabic as well as English in case the respondent felt more comfortable with open ended questions in Arabic

Thank you very much for your comment, we performed this to help even other authors to reproduce similar studies and to help students choose which version is more comfortable with although the medical schools use English in Libya.

• Grammatically beginning multiple statements in a row with “however” loses the reader to what statement we are relating these to. Rewording of the section of “Assessment of medical students’ attitudes toward e-learning” recommended.

I have changed this and removed however, please check with

• A significant number of students (33%) report that the pandemic has affected this career plan or at least their area of interest, while 44% are now interested in infectious disease. This would be a fascinating longitudinal look on if that interest remains in the future.

I agree with you, and hope that future studies that may follow up with more on the changes of the medical students' attitude and career choices toward specialty training that are related to infectious diseases or public health.

Thank you again for your kind revision, we really appreciate your time and consideration.

Best wishes

*************************************

Reviewer D
Review about research article submission: The Impact of the COVID-19 Pandemic on Medical Education: Medical Students’ Knowledge, Attitudes, and Practices Regarding Electronic Learning
Summary:
Alsoufi et al. examine the knowledge, attitudes and practices of Lybian medical students in regard to their experience with electronic learning during the COVID 19 pandemic. The article is a descriptive quantitative study which addresses a timely topic that medical students are facing at a global scale. It gives an interesting perspective about the opinions and challenges faced by medical students in Lybia from 13 different medical schools, including their demographics, social situation, wellness, technological capability and impact of the pandemic.

Strenghts:
The problem statement, conceptual framework and research questions were clear and well articulated. The authors reached out to an important number of students (4500) and utilized several strategies to facilitate the return of answers such as providing equivalent electronic (email and social media), and paper versions of the questionnaire in both English and Arabic in an attempt to secure a good response rate of 74%. The questionnaire was developed based on open ended interviews with students and thoroughly validated internally, with appropriate psychometric properties, in addition to include portions of previously validated tools such as PHQ-2 and GAD-7 for certain areas of interest. Data quality control is described. The data analysis and statistical tests are sufficiently described.
The results of the study are interesting and pertinent to the reality of medical education in Lybia during the pandemic. The literature cited is current and the authors’ claims are framed in current concepts. The authors describe the demographics of medical students in Lybia, their challenges (only 27% participated in online medical education during the two-month period) and their opinions towards e-learning including what they know about it, their practices, and the disruptions it caused in their lives, wellness and studies. For example, it was interesting to see that most students did not rely on their university for their education during the pandemic, but instead pursued self-study educational resources, as most universities suspended their educational programs to certain degree. The discussion proposes several possible interventions to address the challenges identified such the potential of social media platforms as a mean of support or channel for mentorship during this time to address the mental burden of the pandemic.
Specific to the criteria for publication in PLOS ONE, the study is relevant to the mission of the journal. It presents the results of an original research that, to my knowledge, have not been published elsewhere, providing new information on the subject. The statistics and analyses were performed to a high technical standard and are described in sufficient detail with potential to be reproduced. The article is well organized, written clearly in an intelligible fashion and in standard English. The tables are clear and agree with the text. The authors provide the standards for research integrity and data availability.

Thank you very much for your great comment.

I really appreciate your time and efforts to revise our manuscript.

Your kind comment is really great and really helpful for me and I am really happy to see my work getting reviewed in a great way by you.

Thank you so much.

I will try to address any comments below each one

Major comments:
• Due to the unique socio-economic, political and technological circumstances in Lybia, such as the impact of civil unrest or the availability of consistent internet access, it would be optimal if the title would include that this study was performed in Lybia, as its conclusions are likely also unique to their situation and cannot be generalized to other countries or areas of the world. This should also be made explicitly clear in the discussion.

Thank you very much, I have addressed this in conclusion and discussion as added as follows in the discussion to provide a hint that the settings is different and the results may not be generalized and need further studies to validate the tool in more details.

" However, our study was performed in a single country with specific settings. Therefore, the results may not be generalized to other countries, and they must be validated by further studies in different countries and centers to obtain an overview of the utility of the online learning platform as a mode of teaching.”

I think one of the main aims of the study is to provide a repeated tool that can be used in world wide settings, which I mean that we hope that our tool and study is replicated around the world. So, we may think that make it general in title and specified it in the texts might be appropriate as this may lead many readers to focus on the concepts of the study rather than specified it to Libya. However, if the editor agrees, we may add Libya to the title and change it if possible.

• The selection process of whom received the 4500 questionnaires should be described to avoid selection bias.

Thank you very much for your kind comment.

I have added this as follows:

" Students enrolled in these medical schools were selected as follows. In the online version, using Google Forms, a specific question related to medical students’ enrollment status and the name of the school that they attended was used to ensure appropriate selection without recording identifying data. A Google Form containing the study questionnaire was distributed among specific social media groups comprising medical students, or personal emails and messages were sent to then to ensure the appropriate selection of study participants. A friendly reminder was sent to potential respondents to ensure the highest possible response rate. The paper version was distributed among medical students through medical schools and peers. Completed questionnaires were collected in a predetermined place for each school by one of the authors to ensure confidentiality and to prevent any response bias. Unreturned questionnaires were recorded as missing. Participants were not aware of the study aim or outcomes to reduce the risk of any possible bias. The survey included only medical students who were enrolled in Libyan medical schools. The questionnaire was self-administered without intervention by the authors or any specific person, and it did not contain any identifying data of the participants to ensure confidentiality. Questionnaires with incomplete information or missing data were excluded from the analysis.”

• The authors explore the differences in demographic variables between genders. Even though this is an important description that showed that the majority of answers were from female students (71%) and had certain variables with statistically significant differences between genders, I would have liked the authors to explore differences in responses between other sub-groups, such as medical students during their pre-clinical years and clinical years, and the impact of the pandemic on each stage of the education. This is particularly important because the majority of the respondents were from pre-clinical years, and only 13% of respondents were working in a clinical environment, but all the respondents were asked about their opinion on the disruptions on clinical practices, with 54% of them stating that e-learning cannot be applied to the clinical portion of medical education, in which they were not directly involved yet. This observation, in my opinion, needs to be more clearly stated.

Thank you very much for your comment.

Regarding the clinical year, I have added another table for them to make the necessary changes as Table 1B. And the original table as Table 1A.

However, please note that according to the local medical schools, fourth (17.4%), fifth (21.9%), and internship (10.3%) are clinical years, although fourth and fifth have some specific time for clinics and sometimes for usual university lectures. So about 49.5% are from clinical years.

Please see the changes where I combined fourth, fifth, and internship together compared to pre-clinical year and added them as Table 1B.

Thankfully, we found some great difference and discrepancy as stated by the tables.

Also, I have made a note about this in the result section as follows "

“On comparing clinical and pre-clinical years medical students, we found statistically significant differences in age, marital status, gender, health, psychological, physical or learning disability/illness, difference in source of COVID-19 news, and presence/absence of anxiety and depressive symptoms (p < 0.05). Interestingly, health related issues, anxiety symptoms, and depressive symptoms were higher among pre-clinical students as compared to clinical years students.”

I tried to make it shorter and summarized as you suggested, hope this help us.

• The results about the respondents’ understanding of e-learning, the cutoff between adequate understanding and poor understanding was arbitrary. Most of the students had a poor understanding of e-learning (75%) (line 255).

However in the discussion, there are several affirmations that state that students had a high level of knowledge regarding e-learning (line 301,441, 447, 453) , which is a discrepancy with the information obtained in the results. There might be an error in one of those two places (results or discussion).

Thank you very much, for the first one " poor understanding of e-learning (75%) (line 255)." This was based on the tool overall score, for those who scored 5 or more. However, overall, the answers as stated Table 5 states that students are knowing and have knowledge about e-learning.

Also, in line 301, it indicates overall accepted score, therefore maybe using accepted I changed it for better intended meaning

" The results revealed acceptable level…."

This is acceptable although as you said that the cut-off score is somehow arbitrary, therefore, I have made this statement clearer in the discussion.

I have changed 441 into acceptable instead of high

I have changed 447 into variables because it is combined sections

I have changed also the 453 also into the as summary.

• Similarly, the quality of internet service was described as acceptable, good or very good, but only 12% of students believed it can support e-learning in Lybia. Yet, the authors conclude that the study findings support the feasibility of implementing e-learning programs for medical students (line 337-338). The authors do state that downloadable materials may be a better solution than live instruction, even though this approach may not promote interaction.

Regarding the first point, as you can revise the Table 2 which shows the internet types and quality of available of internet services according to students as below, More than half have 4^th^ generation (62.8%) which as we have stated in the discussion along with that 21.5+35.1+29 have stated acceptable, good, very good internet quality which is summarized as you mentioned in line 337-338 " Most reported that they had access to fourth-generation internet services with an acceptable or good internet connection."

| **Type of internet service available (can choose multiple answers)** |  |  |
| --- | --- | --- |
| Asymmetric digital subscriber line (ADSL)  3rd Generation (3G)  4th Generation (4G) | 838  1289  2102 | 25  38.50  62.8 |
| **Quality of internet service**  Bad  Acceptable  Good  Very good | 484  720  1174  970 | 14.5  21.5  35.1  29 |

While regarding the downloadable content, although it may not support interaction but it's a good way of learning for those with low quality internet connection as Libyan have electricity blackout issues and internet cut, therefore that is why Table 6 the participants stated that as we reported in the results section that " However, 56.3% agreed that downloadable video lectures are better than live lectures."

| Attitude | Strongly Disagree | Disagree | Neutral | Agree | Strongly Agree |
| --- | --- | --- | --- | --- | --- |
| **Downloadable E-learning content is better than Live content** | 302 (9) | 469 (14) | 692 (20.7) | 1129 (33.7) | 756 (22.6) |

Because due to internet cut and blackout issues, they would prefer having download option that they can see with any time that is not dependent on stable internet connection or specific places especially that many places outside the capital have internet issues.

• The limitations of the study need to be more explicitly stated.

We have changed the limitations section in the discussion as you can see:

"In this study, we observed that most medical students had access to electronic devices and were able to use them. We also found that medical students displayed variable levels of knowledge, attitudes, and practices regarding e-learning. However, our study performed in a single country with specific settings that the results may not be generalized to other countries, therefore the method must be validated by further studies which could investigate this situation in different countries and centers in order to obtain an overview of the utility of the online learning platform as a mode of teaching, and to determine whether it can replace traditional medical lectures and provide solutions for the disruption that has been caused to clinical training. Another limitation is the cross-sectional study design which means that drawing association need further longitudinal studies in different countries."

Minor comments:
• The data collection tool was provided in the supplementary files. It would be ideal to see a description or script of the open-ended interviews utilized to develop the questionnaire in the supplementary files to promote its replication.

We used several open-ended questions and interviews asking students about e-learning, getting their ideas of the questions that should be asked. The authors do a good job themselves by providing suggestions of questions that some of them are in the questionnaire, while we asked some experts about this in order to reach the questions that should be asked and to avoid unnecessary and controversy questions as possible. We hope to publish the questionnaire to be published along with the study in PLOS One, so many can replicate and be able to publish studies based on this tool, maybe further improvement in future if possible.

• The characteristics of the researchers should ideally be described (are the authors students themselves?) when describing the data collection process.

Not all of the authors are students, while some are already students or final year interns or doctors. However, to avoid any bias especially interview bias, the authors did not collect the paper questionnaire from the participants directly as asked them to put the complete questionnaire in a specific place to avoid any bias, While for the online survey, they sent the anonymous questionnaire to students email and messages, and to the social media groups.

Some details of the data collection process were provided in the method section, kindly please check with the new added section.

• Even though the authors report health and psychological related issues, and the impact of the pandemic is specifically asked, it is difficult to isolate the effects of the pandemic from other socio-economic factors such as the civil war or displacement. The impact of the pandemic on the variables is unclear in comparison to their prior difficulties from financial or social conflicts, and this should be explicitly stated as potential confounding variables.

Thank you very much, you are right and I have made this as an additional statement in the limitation section as follows:

" Another limitation is the cross-sectional nature of the study design, which limited our ability to derive causal associations. This reveals the need for conducting longitudinal studies in different countries. Another limitation of the study, given the specific circumstances of Libyan medical students in terms of the effect of the ongoing civil war, internal displacement, socioeconomical issues, and health-related issues, it would be difficult to separate the isolated effects of COVID-19 on the study variables. These variables may have had a confounding effect on the impact of the COVID-19 pandemic on Libyan medical students.”

• In the discussion, the authors have a strong position about what medical students “should” do with their time during the pandemic, stating that they should pursue volunteering efforts. This is a complex decision with multiple factors that influence it and is not explicitly supported by the data collected in the questionnaire.

You are right, although it is not supported by the data, however we recommended volunteering works as part of the extracurricular activities as we found that many students spend a lot of time in other things, while few are volunteering with only 626 (18.7%) although the pandemic is hitting Libya hard, however the volunteering does not necessary mean working with patients, because many virtual experience can be part of the volunteering activities as we suggest.

• Some of the potential solutions stated in the discussion rely on higher level interventions such as governmental involvement in solving financial challenges, population displacement or improving technological resources, which may not be practically solved in a timely manner.

I totally agree with you to this critical point you mentioned, however as aim to publish our article in a leading prestigious journal, and we will use this published article to advocate and support the medical students in Libya, as this will help to reach high authorities. Also, as a researcher, we just put recommendations as part of the discussion, however using them by the other side as authorities or people might be hard for us. However, we hope that this study will be the first pillar for several following studies and interventions to help mitigate all of these negative effects that are happening to all Libyan medical students.

• The solutions proposed in the discussion should follow a systematic curriculum development approach, including the establishments of goals, the educational strategies, the implementation and the evaluation processes. The discussion only briefly mentions that teachers helped developing plans to achieve the educational objectives of the teaching courses, but it is unclear if they followed a systematic process, or if students who studied independently, for example, followed the same approach guided by the faculty. There are several educational strategies proposed such as interactive online cases, downloaded lectures, or telemedicine, but these need to fulfill specific goals and need to be assessed. Students at different levels of learning have different needs and objectives to fulfill so the solutions cannot be implemented irrespectively of the stage of education.

Thank you very much for your critical comment, I totally agree with you, however as the mentioned efforts are still personal efforts without specific guidance, also many schools are closed unfortunately, and it this moment there was no specific plan or set up goals, so it might be difficult for us to elaborate on this point, however I have added the following sentences in the discussion to mention the points that you kindly mentioned in this comment.

I have written about this in the discussion part as follows:

" However, these proposed learning approaches should follow a systematic curriculum that is developed by experts, and which includes the establishments of goals, educational strategies, implementation methods, and evaluation processes to ensure that the intended learning goals are met. Further, as students at different levels of learning have different needs and objectives, such programs should address students’ needs and goals, as well as they university’s objectives. “

• The process of summative evaluations for decisions about graduation is mentioned in the discussion, but it is not clear how this is done currently in Lybia, if any.

If you mean the graduation from medical schools in Libya. The students have to pass as mentioned in the introduction the essential biomedical year, followed by pre-clinical years and then two clinical years followed by one year of training as intern, then graduation. However, if you think that any point that need to be addressed further, please let me know if possible.

In conclusion, this study describes the knowledge, attitudes and practices of medical students in Lybia during the COVID pandemic, a timely topic with a large sample and many variables investigated. It does provide a good understanding of the situations and challenges faced by medical student in Lybia and the discussion is supported by a good breadth of current literature. The study is very specific to Lybia due to other socio-economic, technological and political situations faced by the country. The difficulties faced by the medical students in Lybia may prevail longer than the pandemic and it is interesting to think how the proposed interventions can become long-lasting. The study proposes further questions and some possible short term solutions. Other long term solutions such as civil, financial, technologic and social problems would need to be addressed simultaneously at a higher level for the proposed solutions to be feasible. If the major comments are addressed, I believe the study does fulfill the requirements for publication stated by the journal.

Thank you very much for your kind revision

I really appreciate your time and efforts to revise our manuscript and your comments helped us to improve our manuscript in order to meet the highest possible standard if possible.

I totally agree with the points you mentioned and that is the main goal of the study, as this study will be the pillar for further studies and interventions that will depends on our results in order to make changes to help medical students in Libya. Also, we hope that our tool be used in other countries and settings in order to provide status of the medical students around the world and to help authorities and high level societies to support students in this hard times of pandemic.

Reviewer G

This national survey of medical students in Libya provides an informative and interesting perspective on how different countries and cultures are responding to COVID in similar and in different ways. The authors acknowledge that this is simple cross-sectional survey and that the study is constrained by that, but the data are very recent and timely and the large sample size is remarkable and the response rate very good for a survey. Libya’s unique situation and recent history can be interpreted as unlike most other countries and thus of limited generalizability, but I believe that the data reflect important underlying attitudes and opportunities for students who like reflect many students throughout the world. Thus, the specifics of the context of this study may be limiting, but the conclusions are worth broad consideration.

The description of the questionnaire development is very detailed and informative. This is particularly important given the use of two languages, so the care taken provides confidence that the results are comparable across questionnaire versions. The use of open-ended questions in interviews is another signal of quality in the methods of this study.

The statistical procedure are reasonable for the data characteristics. In particular, the frequent presence of skewness in the distributions of many items makes the non-parametric Mann-Whitney a good choice.
Thank you very much for your kind comment, I really appreciate your kind words and support.

I really appreciate your time and efforts to help us to revise the manuscript to improve it into the highest possible standards with your great help.

We hope that our study can be replicated in several countries and be able to see future reports based on our findings and tool if possible.

I have tried to address your important comments, kindly check with and let me know if possible.

I have several suggestions that I think will improve the paper:

The purpose of the study (lines 98-100) is clear but I would be interested in the authors’ thoughts about what actions should be taken at a national, institutional, or individual level as informed by these results. How would these data make a difference in the medical education system? For example, I find it striking how much of the students’ education has moved to private courses or open internet resources, away from the individual institutions. This seems striking and to have implications for the future of medical education in Libya (and more generally) – what thoughts do the authors have on such findings?

Thank you very much for your kind comment, I totally agree with you in some extent.

We have proposed several methods and approaches in the discussion part of the study as interactive online cases, downloaded lectures, or telemedicine, with some details from the references provided with some specific experience, also I have added a new paragraph on more details about how these approaches should be and how they can be done in such systematic way as you can see starting from the paragraph : " However, these proposed learning approaches should follow a systematic curriculum that is developed by experts, and which includes the establishments of goals, educational strategies, implementation methods, and evaluation processes to ensure that the intended learning goals are met. Further, as students at different levels of learning have different needs and objectives, such programs should address students’ needs and goals, as well as they university’s objectives.”

However, for national recommendations and actions needed, we have proposed some suggestions and actions that need to be done from the authorities and governmental bodies in the discussion such as you can see in the discussion part starting " Medical students in Libya are facing several challenges……"

For institutional based and also national one, we have proposed several approaches as mentioned above in order to help medical students to tackle these challenges during COVID-19 pandemic and civil war.

On individual level, we tried to provide an overview of the students' status during the pandemic and hope that can help to inform policy makers to take actions and necessary changes.

However, your point of the private practice is really important and I have elaborated this point further per your request, because due to some transport issues, faculty issues as absence of some teachers or their inadequacy of their teaching methods, many students have started to take private courses in order to better understand and to be able to get better way of learning rather than the traditional lectures.

I have made a statement explanation in the manuscript for this finding of the private courses as follows:

" while 56.8% reported that they depended on courses provided by private educational institutions. Interestingly, about one-third of the students depended on university lectures. Student absenteeism at lectures and reliance on private lessons are major concerns for many universities worldwide. These issues can be explained by reasons such as a lack of interest among students and the teaching style, especially the traditional mode of teaching, which requires students listen to monotonous lectures that lack visual stimulation and provides little opportunity for students to engage in discussions. This mode of teaching creates a sense of boredom during lectures and causes students to feel less motivated to attend future lessons . Other reasons include transportation issues faced by students who may be living in another city, and access to multiple tutors in private institutes might be an easier method for leaning.”

Two statements about differences between men and women (lines 168-169 and 174-175) are confusing – I do not know to which variable these differences are attached. Please clarify.

Thank you very much

Regarding the lines 168-169, that was mean difference between age of female and male participants. I have added the word age after, so it will be " The mean age was 21.87 (5.74) years, with a significant mean difference between male and female participants age (p = 0.021)." This is illustrated by the table of characteristics.

Regarding	line 174-175, I have added the word in educational level and several characteristics, so I have changed as follows:

" However, a significant difference was found in the educational level and several other characteristics of male and female respondents. “

Please check with, we are sorry for this confusion.

The sample of students from 13 different schools makes me very curious about differences among those institutions in student attitudes and experiences, but also in how each is responding to the crisis. I realize that the paper is already fairly long but the addition of institutional analyses would be an important extension of this study and should be considered either in this paper or a separate manuscript.

Thank you very much, I agree with you to some extent, however as you mentioned it would need several more tables. Also, as you can see in Figure 1 where it provides an overview of the response rates from the 13 medical schools, also you can see the results section where it said " The University of Tripoli had the highest response rate with 1,199 retrieved questionnaires (35.8%), followed by the University of Benghazi from which we retrieved 448 retrieved questionnaires (35.8%)."

This is because these two are the main medical schools in Libya and the largest one, so comparing with based on medical school may not have major importance as long as Libyan medical students have very similar circumstances and other medical schools are small and they have limited number of responses, which might be difficult to make wide comparison. However, as requested by, we will make the data available and anyone may try to provide further analysis based on the medical school. However, we can make at least characteristics comparison although we believe that we may have similar ones. Please let me know if possible you have any further suggestion as we have added a comparison between pre-clinical and clinical years of school in a separate table.

The paper is dense from many numbers and percentages, which makes if difficult to understand on a single reading. In particular, it would be valuable to have some refence points for interpreting the data, such as the authors provide in the discussion (lines 316-321). If possible to include more of these reference points, it would make the paper easier to understand.

Thank you very much for your kind comment, I agree with you, for the psychological status, as we found several similar papers, we were able to make this comparison with references, however we tried to find some recent papers on online learning, but as the topic is new we did not found similar study to ours to use it for comparison and make references. However, we welcome any kind suggestion in order to make comparison with other studies. We tried to focus on the major numbers in the discussion without repeating all results as it would be difficult for the readers to focus with. Also, we tried to make our personal opinion and suggestion in each aspects of the discussion as much as possible.

Lines 187-190 are duplications of lines 195-198 – if I’m reading it correctly.

Sorry about this mistake, you are right and the 195-198 were removed as it was duplicate of the previous statement.

In lines 251-256 (and lines 289-293), the authors combine 6 or 12 true-false items into a scale value and then select a cut-point to judge an adequate level of performance. This is fine, but reporting only the percentage of learners who reached the single cut-point leaves me wondering about the distribution over the other values. I would suggest that the author report the proportion obtaining each level of these scales so we can get a better sense of the distribution of learner performance.

Thank you very much, I have made this by adding the mean score and SD, also the variance as follows: " the mean (SD) score was 3.6 (1.4), with variance of 1.9, while 813 (24.3%) had an adequate understanding and 2,535 (75.7%) had a poor understanding of e-learning."

The values in the tables should be formatted consistently with one decimal place for all means and percentages.

Thank you very much for your kind comment, I have edited them to have only one decimal place for all values, please check with the changes that were done accordingly.

Thank you for your time and efforts to revise our manuscript.

Reviewer #2: see attached file. seems i have to fill in 200 characters here as well.

Thank you very much, I have copied it here in order to be able to respond to each point below.

**Reviewer Comments:**

I appreciate the timing and value of this study on medical students’ perceptions of online learning in Libya in the context of the ongoing COVID-19 pandemic and the civil war. It is a remarkable testimonial to the commitment and resilience of the students and the faculty that such serious medical education is continuing. Also, impressive that the authors were able to collect and analyze such an extensive set of data under these circumstances. As the authors note in their introduction, the pandemic has disrupted medical education, and it is important for medical educators around the world to adapt to these extenuating circumstances.

There are a couple strengths of the study:

1. The study included more than 10 different medical schools in Libya, a particularly challenging but not entirely unique context for medical education, and they collected data from students across all different class years with a high survey response rate.
2. The survey used in the study included measures across many different domains, allowing extensive triangulation about Libyan medical students’ circumstances and knowledge, attitudes, and practices related to online learning.

Thank you very much for your kind comment, we really grateful for your time and efforts to revise our manuscript in order to meet the highest possible standards.

We hope our study inform medical educators around the world if possible.

Please find my responses below each kind comment.

Overall, I appreciate the findings the authors reported in this study and believe their data provide valuable information about the challenges of adapting medical education in the midst of the ongoing pandemic. However, I would strongly recommend a number of aspects of the analysis and presentation be substantially revised for this study to be considered for publication. These are listed below, roughly in order as they appear in the publication. The most serious issues are in bold. See item #12 in particular as a restructuring of the paper that is needed, and #8 in particular as something that I would see as a rather large but essential change in the analysis.

Thank you very much for your comments, I will try to address them as below each number with red color, please check with and let me know if possible.

1- The authors mention that the survey was distributed to “more than 13 medical schools in Libya” (line 104), but they do not state the exact number in their manuscript. They should report the exact number of medical schools included in the dataset analyzed for the study.

Thank you very much, you can find the exact number in results section as follows " We collected 3,348 complete questionnaires completed by medical students from more than 13 medical schools in Libya." Which is 13, however as there was online survey part of the study, we provided "other option" in case it was not included in our list, however we believe that we were able to cover most of the universities in Libya.

2- There is a very large difference in the number of respondents from the different schools, with three schools having large fraction of the total. It would be useful to break the analysis down between the three large and many small institutions, or at least large and small number of respondents, as I would expect there could be substantial differences between the two groups

Thank you very much, this is due to the fact that these are the main medical schools in Libya, with the largest proportion of medical students in these schools. While, other school have few students as they are based in small cities. However, as previous reviewer mentioned, it would be lengthy to make more tables for comparison in the same manuscript, especially that they are from Libya with similar settings and circumstances, in which we expect similar results between them. However, if the editor agrees, we may make more tables for the comparison as supplementary tables, which might need more space and description in the manuscript.

3- The authors mention that their analysis excluded questionnaires that were either incomplete or contained missing data (lines 106-107). However, they do not report in in the “Results” section how many questionnaires were excluded based on these criteria. The authors should report this number.

They were part of the non-respondents' surveys, as stated that we secured 74% complete questionnaires, while others were non-respondents or incomplete data.

4- The authors provide only an estimated response rate (line 165). The authors should abide by common reporting practices and indicate the exact response rate.
We tried to estimate the response rate based on our estimate or paper sent and messages used, which is a limitation of the study design.

**5- 71.4% of respondents were female and only 28.6% male (line 167). The authors do not provide an explanation or discussion of this dramatic skew towards female participants and whether these percentages are representative of the female/male breakdown of medical students in Libya. Discussion of this skew is particularly important to include given that Table 1 shows that several important characteristics of the study population – for example having financial issues, currently displaced/relocated, and physical or learning disability – are significantly different between females and males in their study population.**
Thank you very much for raising this point, as previous reply to the reviewer, the medical students in Libya are primary female, although there is no official figures and numbers, but based on the local experience, most of the medical students are female, adding to this, they are more active and they usually participate more than male in activities and research projects. Even though we found such discrepancy between male and female medical students. We tried to make this as a note in the statements as there was some associations and discrepancy between male and female in the study.
Therefore, I have made a statement explain this in the discussion as requested " The large discrepancy between the number of females and males participating in the study might be due to the fact that most of the medical students in Libya are female, without official figures or numbers. A second reason is that female students are more likely to participate in research and volunteering activities than are male students”

6- For Figure 1, rather than a pie chart, the authors should report their findings as a table, as per typical reporting standards for the type of data they include in this figure.
Thank you very much, I have made a supplementary table 1 to provide the list of the university participated with their responses number. Please check with.

**7- They list the educational experiences on questionnaire as “Lectures provided by the University. Courses provided by private education centers / courses. Self-study utilizing various educational sources”. While these labels are apparently well understood by the respondents, there should be brief explanations as to what each of these would entail for benefit of international readership. In particular, the label “lecture”, can encompass some quite ineffective pedagogical practices, as well as some effective ones depending on the context and who is using it. I am assuming here they mean only the basic meaning of simple transmission of information via an instructor talking, with no interaction with students or between students. If any of these methods involve more than just basic information transfer and did involve various types of interaction and formative assessment, that needs to be examined in more detail in the analysis. It would be quite misleading to lump together the perceptions of students that experienced such different educational experiences.**

Thank you very much for your comment, you are right, but these lectures meant by that usual lectures of the university, where a tutor provide guidance for their students through explaining the concepts, however it would be difficult to determine each of these lecturers and whether as you said ineffective or not, which may need further studies to inform. Therefore, it would be difficult to determine who is using it to explain for their students and who students have attended these or not because even attending have several issues and some students may find it challenging to attend the usual lectures. Therefore, we cannot confirm if that information transfer method have interaction or not, as this may need further detailed studies.

**8- On line 268, they say that only 27% of the respondents had experienced e-learning. It makes no sense to lump together the attitudes and perceptions of e-learning of the ¾ of the students that have never experienced e-learning with this ¼ that have. In fact, I would argue it is not very important what that ¾ thinks, and it would be much more meaningful to only look at the results of all the e/online learning questions for those ¼ of the students that have actually experienced it.**

Thank you very much for your comment, those of 27% meant that they already starting experiencing e-learning methods, not just online lectures as you can see that many students are using video lectures or internet for studying, but as we mentioned that some department or doctors and tutors are starting to use e-learning lectures with simplified approach, therefore there was some students who tried with, however we cannot generalize that on all other students.

**9- The authors make the conclusion in the “Discussion” section that their study findings indicate adequate knowledge, attitudes, and practice levels to support implementation of online learning in Libya (lines 453-454). I disagree with this conclusion. The authors reported in their “Results” section that 73.6% of respondents believed the quality of the local internet was not sufficient to facilitate online learning platforms (lines 273-274), 66.5% believed conflicts in Libya could pose challenges for online learning (lines 274-275), 78.3% found it difficult to participate in online learning because of financial costs (lines 275-276), and only 20.2% believed that Libyan medical schools could implement online throughout the pandemic (lines 276-277). As the authors describe in their “Introduction” section, the goal of this study was to assess the feasibility of online learning for medical students (lines 96-97). Their reported findings suggest that there will be significant barriers to implementing online learning for Libyan medical students based on the findings described above. The authors’ “Discussion” section does not acknowledge these barriers. I would strongly recommend the authors revise their “Discussion” section to better reflect the findings reported in their “Results” section and provide a discussion of potential strategies that will need to be implemented in order for online learning to be feasible.**

Thank you very much, we have made statement for each point in the discussion, you can check with for example, the financial issues and how should the government intervene, anxiety and depressive symptoms level, also I have added several paragraph that mentioning this in details:
**"**

“Therefore, local governments should provide support for the Libyan population who experience these threats, by providing temporary residence while trying to support their return to their original homes and by increasing security measures in Libya to fight organized crime and activate law enforcement.”

Regarding the support of the implementation, we can see that students needing this way of learning although of these barriers, which should addressed.

Regarding internet issues, you can find in the discussion section starting from "Furthermore, medical students reported high levels of computer and information technology proficiency; about 90% of respondents reported that they had good, very good, or proficient skill levels….."
Regarding the financial issues, I have discussed it in further details in the discussion as follows;

“However, we found that 66.5% thought that conflicts in Libya could pose challenges for e-learning. Additionally, 78.3% of the study participants thought that it would be difficult to participate in e-learning due to financial costs, especially due to the civil war and financial crisis in Libya. These challenges made it difficult for medical students to acquire stable online access, with possible difficulties in using advanced technologies that might be needed in e-learning. These tools and services may be expensive for medical students in Libya, especially considering that medical education is free. Therefore, it is vital to address these issues by providing support to medical students through internet companies by providing a stable and reliable internet service, and by reducing costs for medical students. Faculties and medical schools could support students by providing lectures as downloadable and easy-to-access resources. Further, local governments should facilitate the educational process by providing financial support for students and their family, and by trying to mitigate the negative consequences of the civil war. Additionally, considering the financial implications of the civil war in Libya that would influence all of the above interventions, governments should provide specific financial and information technology support for students to enable them to access low cost and easy-to-use e-learning platforms. “

Regarding " only 20.2% believed that Libyan medical schools could implement online throughout the pandemic (lines 276-277)." This is just report from the medical students' attitude, which is might be true and need to be addressed. However, we just report what we saw from medical students answers to these questions, which might be different in other regions of the world.

" The authors’ “Discussion” section does not acknowledge these barriers. I would strongly recommend the authors revise their “Discussion” section to better reflect the findings reported in their “Results” section and provide a discussion of potential strategies that will need to be implemented in order for online learning to be feasible."
We have addressed most of these barriers in the discussion and proposed several methods and possible approaches to tackle these ongoing challenges.

10- There is lots of discussion at end about possible things to do, but not based on any of their data, just repeating material published and well known elsewhere, without any particular justification that it would work in this context. This does not seem to add anything worthwhile to the manuscript.

we have proposed methods and approaches that might be helpful to tackle the challenges that we found according to the medical students in Libya, and we provided these approaches based on several reports published. For those as psychological status, we have made statement about them and compared with the recent literature. About being these methods working or not depending on the future. In case the responsible authorities or even other countries used our approaches and find solution, then that would be fine. However, we cannot determine that these approaches will work, but we just need to provide our recommendations in the discussion section. But in case of any future approaches, we will be able to address that and measure the effectiveness of these approaches and whether they justify or not.

**11- The “Discussion” section does not include any direct discussion of limitations of this study; the authors only make suggests for future research (lines 448-452). The authors should describe specific limitations of their study and findings such that readers can interpret their findings in context. As part of this, they should also discuss what aspects of their findings could be generalized and to what contexts. In particular, what aspects would likely be relevant to many other countries struggling to provide medical education in the presence of covid and other major civil disruptions, and what aspects are more likely relevant only to the Libya context?**

Thank you very much, I have addressed this part as according to previous comment of other reviewer, you can find the limitations stated in the manuscript starting from " In this study, we observed that most medical students had access……."

Regarding other point " In particular, what aspects would likely be relevant to many other countries struggling to provide medical education in the presence of covid and other major civil disruptions, and what aspects are more likely relevant only to the Libya context?"
You can find this in the same paragraph as stated " However, our study was performed in a single country with specific settings. Therefore, the results may not be generalized to other countries, and they must be validated by further studies in different countries and centers to obtain an overview of the utility of the online learning platform as a mode of teaching. Such replication studies in multiple contexts could help determine whether e-learning can replace traditional medical lectures and provide solutions for the disruption of clinical training. Another limitation is the cross-sectional nature of the study design, which limited our ability to derive causal associations. This reveals the need for conducting longitudinal studies in different countries. Another limitation of the study, given the specific circumstances of Libyan medical students in terms of the effect of the ongoing civil war, internal displacement, socioeconomical issues, and health-related issues, it would be difficult to separate the isolated effects of COVID-19 on the study variables. These variables may have had a confounding effect on the impact of the COVID-19 pandemic on Libyan medical students.”

This statement was in order to provide what should be done after this study and what other should done in their countries based on these findings as most of the countries have similar circumstances in which they have their medical schools closed, however not all school started the e-learning due to some challenges that our study found and they might be able to do so. So, we hope that future studies will depend on our results in order to provide key elements of the medical education status and what aspects that need to be addressed in further details.

**12- A more general comment concerns the focus of the manuscript. It presents a large amount of information but there is little attention as to which is important and/or relevant to covid and their research questions, and which is not. The entire paper needs to be more focused, with better defined research questions and results, rather than such a long tabulation of numbers, some of which are significant in terms of educational methods and policy decisions and many are not.**
Thank you very much for your kind comment, I tried to revise the manuscript further in order to organize it, you can find the track change of these changes. However, as you mentioned that the manuscript provides large amount of information, in which we tried to summarize several aspects of medica education status, psychological status, e-learning and several other challenges to have strong and large paper that will be pillar for future research rather than small focused papers. So, although the paper provides several research questions and answered them in details in some extent. But we believe that would be helpful to organize this project in this way in order to get large results with justified sample size that would be helpful in several ways to policy makers and to the relevant readers

We are really thankful for your kind comments and appreciate your time and efforts to help us to revise the manuscript.
