# Supplementary material for: Impact of the COVID-19 pandemic on medical education: Medical students’ knowledge, attitudes, and practices regarding electronic learning
Source: PLoS One. 2020 Nov 25;15(11):e0242905. doi: 10.1371/journal.pone.0242905 (PMC7688124; doi:10.1371/journal.pone.0242905)
Supplement: Attachment — Submitted filename: Author responses to reviewers October 2 2020.docx [file pone.0242905.s007.docx]

Dear Dr. Yuka Kotozaki

Academic Editor

PLOS ONE

Thank you very much for this great opportunity to revise our manuscript in PLOS ONE

PONE-D-20-22931R1
Impact of the COVID-19 pandemic on medical education: Medical students’ knowledge, attitudes, and practices regarding electronic learning

We really appreciate your kind consideration, and we will try to address reviewers and editorial comments with a red color as indicated under each point.

Review Comments to the Author

Reviewer #1: I cannot determine if the data are being made available to meet criteria #4. Everything else seems fine to me.

Thank you very much for your kind comment, I have included the data set in our previous review as zip file that contain raw data in SPSS, if you could check with, that would be greatly appreciated.

We really thankful for your great efforts to revise our manuscript.

Best regards

Reviewer #3: This paper describes the large scale surveying of medical students in Libya concerning their knowledge, attitudes and practices with E-learning. While the challenges the students face are daunting and worthy of attention, it is unclear how the knowledge generate by the researchers would be utilized by a broader audience. While administrators and instructors at Libyan medical schools would benefit from this information, the Journal PLOS One has an international audience and the authors have not made a compelling case for how the results may serve a broader community.

Thank you very much for your kind comments, we really appreciate your kind efforts to help us to improve our manuscript through your valuable comments.

We have made the study initially as generalizable and we share this as single experience, however the tool and scale that we use during the survey is general and can be applied for other countries and settings as we stated, and the knowledge, attitudes and practices with E-learning were general and not attributed to Libya. However, as you could see with the previous reviewers comments as requested to add several examples and specific paragraph to denote that the results are for local, although we made this study as general as we can and we only included some specific variables that are of local concerns as civil war effects. However, we stick to the general idea of e-learning, which many developing countries with similar settings that need to be addressed. Also, we believe that our study will be a pillar for further other international and regional studies that use the same tool to address the concerns about e-learning in medical schools given the upcoming second wave of COVID-19.

Therefore, we believe that our results and method are generable and hope to be replicated in further countries, especially of those countries where e-learning has not started yet, or those countries where there was a severe disruption of the medical education process.

We believe that some few factors are locally, which we admire during our limitation section, however the main message and idea is broader and need to be explore in other countries in further studies.

You could check with the discussion section, where we tried to make general statement about the issue of e-learning. Also, as you could check with our previous response to reviewer comments about making the results and scale more general for broader audience which PLOS ONE support for other international and regional settings.

This concern is further exemplified by the Discussion section which often makes general statements that cannot be directly related to the results. Many of these statements are agreeable and good suggestions, but would likely have been advocated regardless of the survey results. Examples of this include using social media to motivate junior medical students (line 359), providing temporary residence to those displaced and increasing security measures (lines 372-374), providing financial and technological support to students (lines 397-401) and medical students volunteer services (lines 439-440). Similarly, the discussion on low attendance to lectures and the advocacy for interactive discussions on pages 27 and 29 are sound recommendations based on past literature but appear unrelated to the survey results presented herein. Within the discussion, it was not possible to find a clear course of action that directly resulted from the research findings.

Thank you very much for this great and detailed comment.

We tried to provide additional notes and recommendation through comprehensive discussion and we tried to address and discuss further points of medical education during COVID-19 that may not related totally to the aim of the study, as we tried to make a summary and review of the available data and ideas and how can be utilized during COVID-19 pandemic.

Therefore, we tried to make these broad statements to support the study as being a summary of e-learning, along with providing additional good suggestions that were some of them addressed in letters or small studies in the literature, so our study can summarize these findings and ideas for broader medical education audience.

We also tried to provide some possible solution and ideas for some of the issues and concerns that we found during our study. However, as we stated during the previous review round, that these solutions may not be applicable as soon as possible. However, we tried to make some notes for policy makers here and internationally about the struggles and issues that face medical students especially in countries with limited resource settings. And how these challenges and issues can be addressed further.

Therefore, in the discussion section, we tried to provide big picture of e-learning for medical students. Also, we tried to shed light on some of issues and challenges of medical education during COVID-19. Thus, our article provides broad idea and cover major issues and possible previous and current challenges which we mentioned. For example, the idea of attendance, in which we found students not prefer and we provided possible reasons according to our experience and based on limited literature knowledge.

The idea of displacement and civil war effect was addressed in more details as this related to locally and similar countries with similar civil war and conflict issues that need to advocate for students who are suffering from these issues and how can be addressed further. The technology limitation is of concerns also, as stated previously due to the issue of financial and infrastructure which need to be addressed in order to implement e-learning among medical schools, and how this idea can be successful and replacement of usual lectures during COVID-19 pandemic.

More specific concerns:
1) Page 6 describes the survey methods of using both online and paper forms to collect surveys anonymously. Is there the possibility that a participant answered the survey twice, once using each type of form?

# Well this a good question, however we think that students may not do that. Also, during data cleaning, we removed duplicate responses or those responses that were recorded at the same time from online version with same results or those with same results and answers to avoid this issue during data collection to ensure highest possible data quality. Due to anonymously, we did not use specific name or email during survey filling to secure the confidentiality of information collected.

# 2) Page 7 describes the PHQ-2 instrument as "has been validated". Validity requires a consideration of not just the instrument but the intended sample that the instrument will be given to and the administrative procedures used for the instrument (e.g. online or in person, timed or not timed). The authors should comment on the extent their sample and administration matches the prior efforts to seek validity evidence for this instrument.

# We used PHQ-2 instrument and we meant by has been validated in previous studies as a well-known tool for depressive symptoms. And was used in many previous studies, however in response to this comment. I have made a comment on the validity of both PHQ-2 and GAD-7 by calculate level of internal consistency, as determined by a Cronbach's alpha as follows:

# "PHQ-2 scale had a high level of internal consistency among our study participants, as determined by a Cronbach's alpha of 0.8."

# "GAD-7 scale had a high level of internal consistency among our study population, with a Cronbach's alpha of 0.91."

# These two sentences were added in the method section after I calculate the internal consistency using the full population of the study for both tools GAD-7 and PHQ-2. Although we used a validated version and therefore, we did not translate. 3a) Page 8 introduces statistical tests that will be used to compare groups. There is no mention in the introduction or problem statement that a comparison between groups would be utilized in this work. Having the first mention in the methods causes confusion as to why such a test is needed.

We used them as secondary outcomes, as you aware that our study provided several outcome measures and we intended to make them in one strong paper than to divided them into several papers. Also, these comparisons were requested by a previous reviewer. Therefore, we make these comparison just to have an extra outcomes and see if there is any difference based on gender or being pre-clinic and clinical student.

3b) Pages 10 and 11 present the comparison between gender and clinical vs pre-clinical groups. Why are these comparisons conducted? In particular, before conducting the tests, what would observed differences lead one to do versus finding no evidence of differences between these groups?

We performed these comparisons to see if there are any differences in study characteristics based on gender. So, we added them to the table of characteristics. However, one of the reviewers asked to make another comparison with pre-clinic and clinical group of students, therefore, we added another extra table. These are univariate analysis and just to make a note of how the sample of the study distributed. We preformed the gender difference especially that most of the study participants are female as it might it be of importance to check with. However, we added extra table for year of study based on previous reviewer recommendations.

4a) A common phrasing used throughout this work is the word "majority" or "most of" when describing portions of the sample that represent less than 50% of the sample. Examples include lines 188 and 218. These descriptors should only be used for groups representing more than 50% of the sample.

Thank you very much for your comment, I have changed them as follows:
I replace line 188 of majority with >>> Greater number
I replace line 218 of most of >>> Larger number of
For line 218, it was intended to describe both value 47.5% and 19%

4b) Another transition word commonly used is "Additionally" which may lead the reader to think that the percent described is added onto the percent used in the previous sentence. Lines 247 and 250 are examples of this. Recommend avoiding the word "Additionally" when transitioning from one percent value to another.

Thank you very much, I have removed them as requested in line 247 and 250 line and other lines too.

5a) Pages 18 and 19 describe using the data in Table 5 as a measure of proficiency of E-learning. Instead, these statements appear to represent participant's perceived experiences with E-learning. For example, the statement "One of the benefits of E-learning with live content is that the scholar receives instant feedback from the instructor". It seems possible that a participant rates this false because it has not been true in their experiences with E-learning rather than interpreting it as a lack of knowledge about E-learning. It is clearly not universally true of E-learning experiences.

Thank you very much for your comment, this does not necessary that they have experienced this. Because this based on perception of e-learning experience. However, we have added I don’t know for those who did not use previous online learning tool, which does not need to be officially one, but as MOOC courses or any other modalities. That is why we added the option of I don’t’ know. We think that the survey tool might need more improvement, and we wish that we can validate it in further studies in other countries. The Table 5 measure the knowledge about e-learning in general not specifically for medical education and contains statements that provide some insight about any previous experience with e-learning.

5b) A similar concern arises on pages 22 and 23 where students' E-learning practices are summed to determine if they are adequate or inadequate. Some of the statements are also problematic for this measure. Students may not have purchased a device for E-learning because they already owned a device; not because they are inadequate in practicing E-learning.

We agree with you, however as the e-learning device can be tablet, computer, or even any other related device. And we intend to measure the practice, so those who bought device for e-learning or own one, that mean that they are having better experience with e-learning. It doesn’t mean that they don’t own or just bought for e-learning, but owning a device is a positive response.

===============================================================

Reviewer #4: I note that this manuscript has `undergone extensive revision in response to extensive reviewer feedback. I am mindful that there are in fact few studies published that capture meaningful medical educational research in resource poor countries.

Thank you very much for your kind review. We really appreciate your time and consideration to help us to improve our manuscript.

I have tried to address your comments below each one.

The overall research questions that you asked is “we aimed to provide an overview of medical students circumstances during the pandemic, and to determine their knowledge, attitudes, and practices pertaining to digital medical education.” However it appears from the paper that there are a range of data that are presented including wellbeing/mental health, COVID knowledge and attitudes, technology availability, impact of COVID om the quality of medical education, snapshot of how students are spending their days, and understanding, attitudes and current practices in eLearning. I believe it important to separate out what is useful evaluative data locally in Libya and what is of interest to the international readership of the journal. Accordingly, the research aim should be operationalised as the key research questions that the researchers wanted to answer. The literature might need tweaking depending on the prioritised research questions e.g. include some of the Libyan papers on student mental health. (In fact, you pick these up in the discussion, which I think is a little late given the data you have). This would then better link the research questions to the results and the interpretation of them.

Thank you very much for your kind comment.

We tried to make this paper as comprehensive in one paper that capture several outcomes together to have a strong paper with several outcomes rather than separate small ones.

We provide some few local related outcomes such as related to civil war situation for medical students. In addition, the main and primary outcome of e-learning was extensively explained and provided some discussion on the main points, which is the main and generalizable outcomes of the study, of which this outcome was general and is applicable to other countries as we stated.

We tried to provide a tool that may need more validation and further assessment in other countries and settings for comparison which we acknowledge in our section of limitation and discussion part. These e-learning results are of importance to the current literature given the situation of COVID-19 and the upcoming of second wave of this pandemic.

Regarding mental health students’ papers, there was no previous studies on mental health of medical students that were published previously except of one study that we have under publishing that was done in early phase of pandemic before the current study, which found similar findings despite using PHQ-9 in previous study.

I have added this in the discussion

“A previous study performed among Libyan medical students during the early phase of the COVID-19 pandemic, found that 11% of medical students have anxiety symptoms, 21.6% have anxiety symptoms, and 22.7% have suicidal ideation which is similar to our current findings”

Reference

Elhadi, Muhammed, et al. "Psychological impact of the civil war and COVID-19 on Libyan medical students: a cross-sectional study." Frontiers in Psychology 11 (2020): 2575.

In terms of describing the research context, in the Libyan what is the system of medical education funding, student pays, government pays, mixed picture? Are they all five 6 year courses?? Any international students?? Traditional pre -clinical clinical divides?? When students have qualified is there an internship, then specialty training. Do most migrate overseas, or are they required to serve for so long in Libya.

Most of the medical schools are the same system where government pays and the education is free of charge. They are the same system of 6 years with one internship, although some provide 5 years with one-year internship.

They are international students, and there was a question about nationality but we removed it to prevent any specific discrimination for other international students.

They use the traditional clinical and pre-clinical courses.

When students have completed their studies, they have to pursuit to finish the internship in order to get final diploma and certificate degree.

They use the same English system as Britain, India, Egypt….

Then specialty training.

Most of them are seeking migrate overseas for training and post-graduate qualifications. However, many may remain here due to some financial restrictions or personal issues in Libya that may prevent them to migrate overseas. Therefore, many are staying here and continue to work here in Libya. There is no official figures, but this is based on my personal experience.

Given the University of Tripoli had the highest response rate with 1,199 completed questionnaires (35.8%), it suggests the overall response rate with the number of students as the denominator is around 35% rather than the 74 claimed?? Is this addressed in the limitations section? Most surveys of this type suffer from low response rates, but it is the best we have.

Thank you very much. Because University of Tripoli is the largest medical school and majority of Libyan are studying at either University of Tripoli, University of Benghazi, and AlZawia University.

While other medical schools are small and contain very few students, which is another issue to be discussed for policy makers as Libya is large country in term of geographical area, therefore, some small cities have their own medical schools which have few students in their, which explain the low number of responses gathered from other places.

Therefore, we think that we gathered a balanced number of response from overall medical schools in Libya despite that most of them are centralized in specific schools which have this impression of number differences.

Given the number of analyses, the chances of findings by chance are increased. What are the main analyses for the key research questions? This suggests a more theoretically driven analysis rather than exploring possible relationships among variables? and overclaiming findings of statistical significance.

Thank you very much for your comment.

We have provided many outcomes in order to have a solid paper with different outcomes rather than smaller ones. We addressed many issues. However, the main outcomes were on knowledge, attitudes, and practices regarding electronic learning. Which was addressed in specific details given the newly designed tools. In addition, we tried to address other issues as mental health, student’s situation, activities during COVID-19, COVID-19 effect, and civil war effects.

We tried to be descriptive as much as we can. However, we provided some association with study characteristics based on gender and year of study status.

However, the main idea was to focus on e-learning and how we can discuss the challenges and situation during COVDI-19 pandemic rather than specific advanced analysis. However, we are welcome any help with any suggestion within the data that we included with the study.

Out of interest in Table 1B do you think that some of the differences might be that the pre-clinical have no networks, whereas the clinical at least had the chance to make friends and networks?

Might be a reason behind this, although I have made this table in accordance to a previous reviewer comment. But I do think that preclinical are more stressed and have more anxiety and depressive symptoms might be due to fear of medical school or might be due to fear of future or how to deal with many overwhelming basic science subjects. However, your kind opinion is another reason possibly due to friends and network and may exposed more to hospital and patients settings which might relieve this uncertainty about medical school.

In Table 2 many of the percentages don’t add up to 100%, so e.g. how do you know if a student is/is using all three a laptop, a smart phone and a tablet?? Similarly, that most students are using digital devices for both education and social media purposes?

Thank you very much for your kind comment.

I have fixed one which was based on nearing the number, while others have the ability to choose multiple answers together, therefore they don’t add up to 100%. Please check with if possible. I have made a note for these questions that can have multiple answers.

Table 3 for international audiences, be helpful if you define what you mean by suspended. The table suggests, normal teaching has been suspended, but students are still enrolled? It suggests at first reading several programs have been stopped. In fact, you define this in the impacts of COVID section. As suggested by a prior reviewer institutional differences would be important here, but you may not have the data.

Thank you very much for your comments.

I have added this in the beginning of the paragraph to make it easier for audience to understand.

“Medical schools have suspended the educational process due to the COVID-19 pandemic. However, when students asked about their current enrollment status and whether they suspended or paused their education due to any other causes, we found that….”

We meant by suspending that students are currently may not enrolled in a medical school for example, they may pause their study due to civil war, due to personal financial or social issues. Therefore, we asked students if they suspended personally their education due to these causes other than the pandemic, in which all Libyan medical school have stopped and suspended the usual educational process.

We meant by enrollment that active status in the medical school not suspended (not currently students due to personal or any other cause).

Similarly, with how students are spending the time, should you order by percentages, so that the data is clearly showing students watching TV and reading books. Does this mean in addition their studies and in their free time? Or does it mean what they are doing because they are suspended? Might the day appear unfair to medical students otherwise?
Would colleges take a different attitude to suspending courses if they knew that medical students could do both.

Thank you very much for your comment.

I have re-ordered them as you suggested. Please check with if possible.

Due to lockdown of COVID-19 pandemic, as schools are suspended, we just asked these questions if students are doing any of these activities during COVID-19 lockdown.

I totally agree with you that the day might appear unfair. And I agree with the point of doing different attitude, instead of suspending schools to find another solutions to solve this issues instead of students spending their precious time waiting for unknown and uncertainly.

Please check with the tables table 3 as I organized them according to ascending percentage.

Table 4 does require its own research questions. Out of interest this COVID impact scale may well have factor analysed into different constructs??

Thank you very much for your suggestion.

Regarding Table 4 was about medical student attitude toward COVID-19 pandemic. As we stated about this in our research question and provided this table to answer the impact of COVID-19 pandemic on social and mental wellbeing. We may factor analyze into different construct after discussion in following studies.

Table 5,6 and & 7 required its own research question. I though given the number of tables, table 5 could be deleted without loss to the paper. Had you considered showing as bar charts for some variation?? Otherwise the reader has to add up mentally to rapidly digest the meaning of the table.

Sure, this is the main research question of the study, which are illustrated in Table, 5, 6, and 7, which is “determine their knowledge, attitudes, and practices pertaining to digital medical education.” This is the main outcomes in the study and we left it in the end of the results section as the previous one is related to COVID-19 effects.

We tried to make them three tables to make it easier to follow and to provide this as a future tool for other researchers to use after us to determine the e-learning knowledge, attitude and practice during COVID-19 pandemic.

Bar charts might be difficult to use especially that we need to show each of the questions which might disrupt the figure. Table 5 is important as it provide information about the knowledge toward e-learning which is the core of the study. Many were not having adequate knowledge as displaced in response rates to knowledge about e-learning.

We tried to make the tables as focused as possible, and these questions were used after we removed several ones through interviews and discussions to reach this version, which might benefit from further studies on this topic.

In discussion what are workforce implication on having enough qualified doctors in a few years’ time, because of all of this?? For table 7, it is more the evaluation of e-learning practices, rather than them being assessed for their e-learning skills.

I have edited the table 7 and added Evaluation word. We tried to evaluate their practice if someone has used either types of e-learning or even MOOC courses, as there are many international companies that provide medical education lectures and sessions online as local ones does not, and many students are using these external sources. So we added this table to evaluate the practice of e-learning and how students if they used it and how experience it.

Regarding the discussion of workforce implication on having enough qualified doctors is another issue here in Libya that will need further studies, as there is an issue in post-graduate learning process which needs further discussion for the obstacles and challenges that are present.

The discussion is largely about an interpretation of the results, there may be some repetition here from the actual results section. It would be useful to have ta sub heading of implication for policy and practice. However as noticed by a previous reviewer, the implications are not all grounded in the data the authors have reported. New literature around tele health and virtual learning is introduced. It raises the question of this being an area of further research, rather than an afterthought of what might have been included in the current study. The strengths and limitations section would benefit from a sub-heading, with some emphasis on the value of the study.

Thank you very much for your kind comment.

We tried to summarize the findings and some way we focus on main findings and avoid repetition as much as possible. Also, we tried to make comprehensive discussion and provided many aspects for future research area by providing new literatures and ideas of further research. Also, we tried to make our discussion provide as a review article to summarize the current issues and challenges for medical education during COVID-19 pandemic.

I have added sections as limitation and implication for policy and practice.

Thank you again for this great opportunity to revise our manuscript to help us to reach the highest possible quality.

We really admire your help and efforts

Best regards
